# Metagenomic Insight into Microbiome and Antibiotic Resistance Genes of High Clinical Concern in Urban and Rural Hospital Wastewater of Northern India Origin: a Major Reservoir of Antimicrobial Resistance

**DOI:** 10.1128/spectrum.04102-22

**Published:** 2023-02-14

**Authors:** Absar Talat, Kevin S. Blake, Gautam Dantas, Asad U. Khan

**Affiliations:** a Medical Microbiology and Molecular Biology Laboratory, Interdisciplinary Biotechnology Unit, Aligarh Muslim University, Aligarh, India; b The Edison Family Center for Genome Sciences and Systems Biology, Washington University School of Medicine, St. Louis, Missouri, USA; c Department of Pathology and Immunology, Washington University School of Medicine, St. Louis, Missouri, USA; d Department of Molecular Microbiology, Washington University School of Medicine, St. Louis, Missouri, USA; e Department of Biomedical Engineering, Washington University in St. Louis, St. Louis, Missouri, USA; Nanjing Agricultural University

**Keywords:** antimicrobial resistance, metagenomics, resistant markers, waste water

## Abstract

India is one of the largest consumers and producers of antibiotics and a hot spot for the emergence and proliferation of antimicrobial resistance genes (ARGs). Indian hospital wastewater (HWW) accumulates ARGs from source hospitals and often merges with urban wastewater, with the potential for environmental and human contamination. Despite its putative clinical importance, there is a lack of high-resolution resistome profiling of Indian hospital wastewater, with most studies either relying on conventional PCR-biased techniques or being limited to one city. In this study, we comprehensively analyzed antibiotic resistomes of wastewater from six Indian hospitals distributed in rural and urban areas of northern India through shotgun metagenomics. Our study revealed the predominance of ARGs against aminoglycoside, macrolide, carbapenem, trimethoprim, and sulfonamide antibiotics in all the samples through both read-based analysis and assembly-based analysis. We detected the mobile colistin resistance gene *mcr-5.1* for the first time in Indian hospital sewage. *bla*_NDM-1_ was present in 4 out of 6 samples and was carried by Pseudomonas aeruginosa in HWW-2, Klebsiella pneumoniae in HWW-4 and HWW-6, and Acinetobacter baumanii in HWW-5. Most ARGs were plasmid-mediated and hosted by *Proteobacteria*. We identified virulence factors and transposable elements flanking the ARGs, highlighting the role of horizontal gene transmission of ARGs.

**IMPORTANCE** There is a paucity of research on detailed antibiotic resistome and microbiome diversity of Indian hospital wastewater. This study reports the predominance of clinically concerning ARGs such as the beta-lactamases *bla*_NDM_ and *bla*_OXA_ and the colistin resistance gene *mcr* and their association with the microbiome in six different Indian hospital wastewaters of both urban and rural origin. The abundance of plasmid-mediated ARGs and virulence factors calls for urgent AMR crisis management. The lack of proper wastewater management strategies meeting international standards and open drainage systems further complicates the problem of containing the ARGs at these hospitals. This metagenomic study presents the current AMR profile propagating in hospital settings in India and can be used as a reference for future surveillance and risk management of ARGs in Indian hospitals.

## INTRODUCTION

Antimicrobial resistance (AMR) is a global public health emergency. In 2019, an estimated 5,000,000 deaths were associated with bacterial AMR, exerting the highest burdens in low- and middle-income countries (LMIC) (1). Exacerbating the problem, a rise in AMR has been accompanied by a reduction in the number of new antibiotics approved for human use (2, 3). The World Health Organization’s “One Health” approach emphasizes the close association of human, animal, and environmental health (4). Reliable and accurate surveillance is critical for characterizing the risk of AMR in a given region, tracking the spread of specific antibiotic resistance genes (ARG) geographically and over time, identifying new ARGs, and supporting preventative measures and interventions against multidrug-resistant (MDR) pathogens.

Hospitals are important reservoirs and vectors of AMR, where the frequent and persistent use of antimicrobials selects for MDR pathogens that can cause hospital-acquired infections (5, 6). Hospital wastewater (HWW) receives liquid medical waste, and the excrement of patients, visitors, and health care professionals, and as such, contains hazardous chemicals, pharmaceutical residues, and human pathogens (7). In turn, it contains a greater diversity and abundance of ARGs than other wastewater systems (8). Additionally, in many settings, particularly in LMICs, HWW is released into municipal sewage systems without treatment (7). This, coupled with open drainage systems and inefficient wastewater treatment plants, can lead to the dissemination of HWW contents, including antimicrobial residues and MDR pathogens, into the local environment and community (9). Metagenomic surveillance of HWW using short-read next-generation sequencing data is an efficient approach for assessing the overall AMR burden in a given hospital (10–12). Unlike traditional culture- and PCR-based approaches, which are laborious and limited to selected taxa and ARGs, metagenomics can quantify thousands of species, ARGs, and virulence factors (VFs) from a single sample at a relatively low cost. Further, surveillance of sewage instead of samples from human patients has the additional advantage of being easily obtained and analyzed without ethical review. However, current research on AMR in HWW has mainly focused on high-income countries, with limited data reported for LMICs (13).

India is one of the largest consumers and producers of antibiotics and is a hot spot for AMR (14). Antibiotic usage in hospitals in Asia is comparatively higher than in European hospitals (15). A study based on data on patient-level antibiotic consumption from 209 surveys and 284,045 children (aged <5 years), collected over 19 years and covering 101 countries, reported a dramatic surge in the median national antibiotic usage in India from 48% in the year 2000 to 67% in 2018. The lack of awareness about judicious antibiotic usage and the detrimental consequences of “over the counter” medication has caused a notable increase in the consumption of fluoroquinolone and third-generation cephalosporin in India from 2000 to 2018 (16). According to the WHO, by the year 2023, 60% of the total antibiotic consumption in a country should be constituted by WHO-Access group antibiotics, the antibiotics that are efficacious against commonly encountered susceptible pathogens, and there are low chances of resistance development against these antibiotics. In 2015, only 30% of the total antibiotic consumption was covered by the WHO-Access group of antibiotics in India (17). Several factors favor the spread of AMR in Indian hospitals such as over-the-counter drug availability, low doctor-to-patient and nurse-to-patient ratios, antibiotics prescription by informal health care providers, and lack of infection prevention and control guidelines (18–20). India is also the hub for the manufacturing and distribution of generic antibiotics for global use, and this industrial-scale manufacturing and its associated waste likely further contribute to the development and spread of antimicrobial resistance (21). The high usage of antimicrobials in Indian hospitals undoubtedly selects for AMR. However, there are limited data on the ARG burden in Indian HWW, with previous reports limited to select ARGs and culturable bacteria and/or to a single city (22, 23).

In this study, we use shotgun metagenome sequencing to characterize the microbiomes and resistomes of HWW from six hospitals in five cities across northern India. To the best of our knowledge, this is the first study from India, comprised of samples from rural and urban hospitals, all of which were equipped with bed facilities and admitted severe infection cases. Our findings suggest that the diversity and abundance of AMR in Indian hospitals are greater than previously thought, which poses an immediate potential risk to patients and the surrounding communities.

## RESULTS AND DISCUSSION

### Hospital wastewater sample collection.

**FIG 1:**
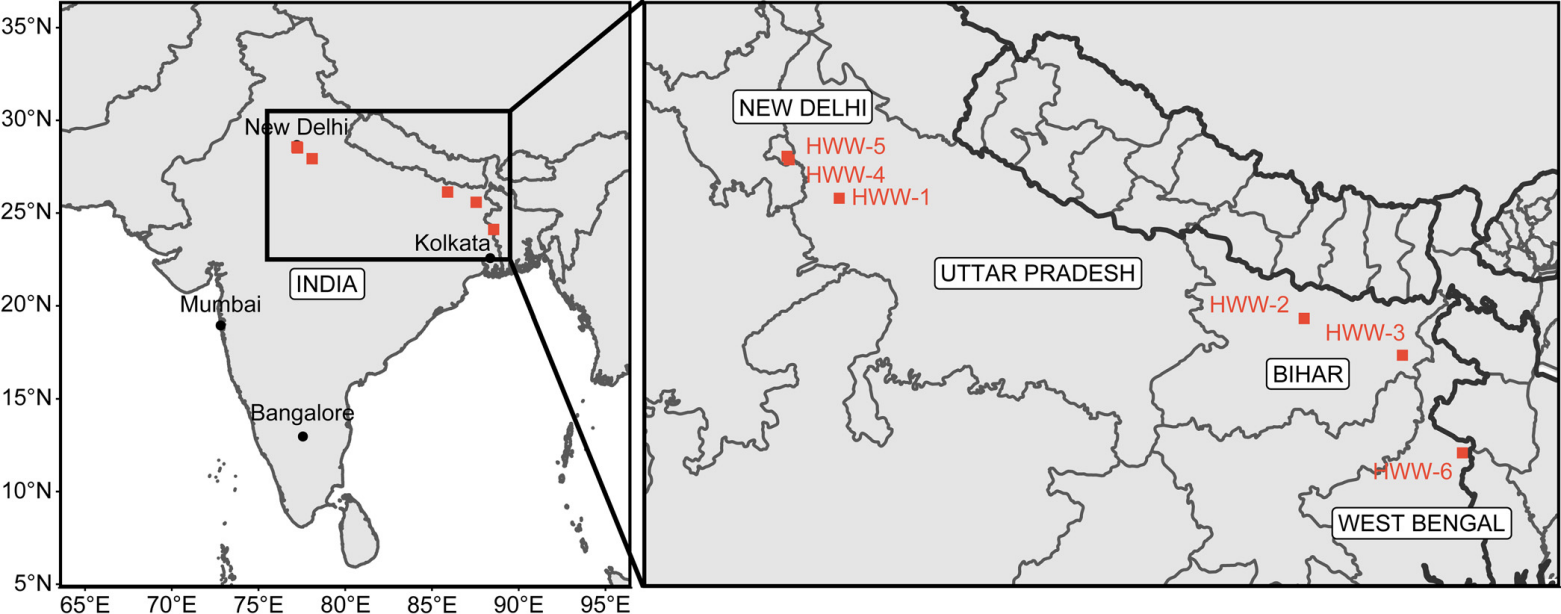
Sampling of hospital wastewater across northern India. HWW-1, HWW-2, HWW-4, and HWW-5 are located in urban areas whereas HWW-3 and HWW-6 are rural hospitals. Due to cost-effective treatment and easier access to HWW-1, HWW-2, HWW-3, and HWW-6, the maximum patient load in these hospitals is from rural areas with below poverty line patients. At HWW-4, both rural and urban populations from rich as well as poor backgrounds come for treatment. HWW-5 has more patients from urban and affluent areas.

Wastewater samples were collected from six tertiary care hospitals in regions of northern India (Fig. 1), with a range of 300 to 2,400 beds (Table 1). The socioeconomic status of the surrounding population also varied at each site. Sample HWW-1 was collected from Jawaharlal Nehru Medical College and Hospital (J.N.M.C.H.), Aligarh, Uttar Pradesh; HWW-2 was from Darbhanga Medical College and Hospital (D.M.C.H.), Darbhanga, Bihar; HWW-3 was from Katihar Medical College and Hospital (K.M.C.H.), Katihar, Bihar; HWW-4 was from Hamdard Institute of Medical Sciences and Research (H.I.M.S.R.), New Delhi; HWW-5 was from All India Institute of Medical Sciences (A.I.I.M.S.), New Delhi; and HWW-6 was from Domkal Super specialty and subdivisional Hospital, West Bengal. All the hospitals except H.I.M.S.R. are government hospitals with cost-effective treatment. J.N.M.C.H. and D.M.C.H. are located in cities, H.I.M.S.R. and A.I.I.M.S are located in a metropolitan city, whereas K.M.C.H. and D.S.S.H. are located in rural areas. The socio-economic data of patients visiting these hospitals were not retrieved, but according to the 2011 census, the slum population in Aligarh and Darbhanga is approximately 30% and 16%, respectively (24). The population living in villages comprises 91% and 80% population of Katihar and Murshidabad, respectively (24). New Delhi has a predominantly urban population (24). An inexpensive treatment and easier access to these hospitals facilitate a high influx of low-income patients at these hospitals.

**TABLE 1:** The information about six hospital wastewater samples collected and analyzed for this study

Sample collection details	HWW-1	HWW-2	HWW-3	HWW-4	HWW-5	HWW-6
Site of collection	J.N.M.C.H, Aligarh	D.M.C.H., Darbhanga	K.M.C.H., Katihar	H.I.M.S.E.R.	A.I.I.M.S.	Domkal Super Speciality and Subdivisional Hospital
State/union territory	Uttar Pradesh	Bihar	Bihar (bordering West Bengal)	New Delhi	New Delhi	West Bengal
No. of beds available	2,400	1,050	590	650	710	300
Collection date and time	21 Dec 2019 at 11 a.m. Indian Standard Time (IST).	10 Jan 2020 at 4 p.m. Indian Standard Time (IST).	19 Jan 2020 at 2 p.m. Indian Standard Time (IST).	29 Jan 2020 at 12:40 p.m. Indian Standard Time (IST).	10 March 2021 at 10:50 a.m. Indian Standard Time (IST).	21 March 2021 at 5.30 a.m. Indian Standard Time (IST).

Wastewater samples were collected from the main hospital sewage water pipeline, which receives the effluent of the entire hospital. Samples were processed and submitted for shotgun metagenomic sequencing (Table 2).

**TABLE 2:** The classification of reads by Kraken2, visualized in Pavian v1.0

Sample	No. of raw reads	Classified reads	Unclassified reads	Chordate reads	Artificial reads	Microbial reads	Bacterial reads	Viral reads	Fungal reads	Protozoan reads
HWW-1	2,67,48,085	44.56%	55.44%	4.99%	0.00%	39.34%	33.29%	0.19%	0.78%	0.22%
HWW-2	2,17,73,844	49.79%	50.21%	13.19%	0.01%	29.86%	22.09%	0.14%	0.94%	0.31%
HWW-3	2,21,34,911	47.24%	52.76%	8.93%	0.00%	38.08%	7.53%	0.25%	1.77%	0.77%
HWW-4	3,72,83,617	69.08%	30.92%	3.59%	0.00%	65.07%	60%	0.22%	0.58%	0.16%
HWW-5	2,03,38,684	59.81%	40.19%	4.38%	0.00%	55.24%	49.83%	0.12%	0.77%	0.15%
HWW-6	1,97,81,363	56.56%	43.44%	4.73%	0.00%	51.62%	45.44%	0.21%	0.80%	0.42%
Average	2,46,76,751									

### Dominance of *Proteobacteria* in HWW microbiomes.

**FIG 2:**
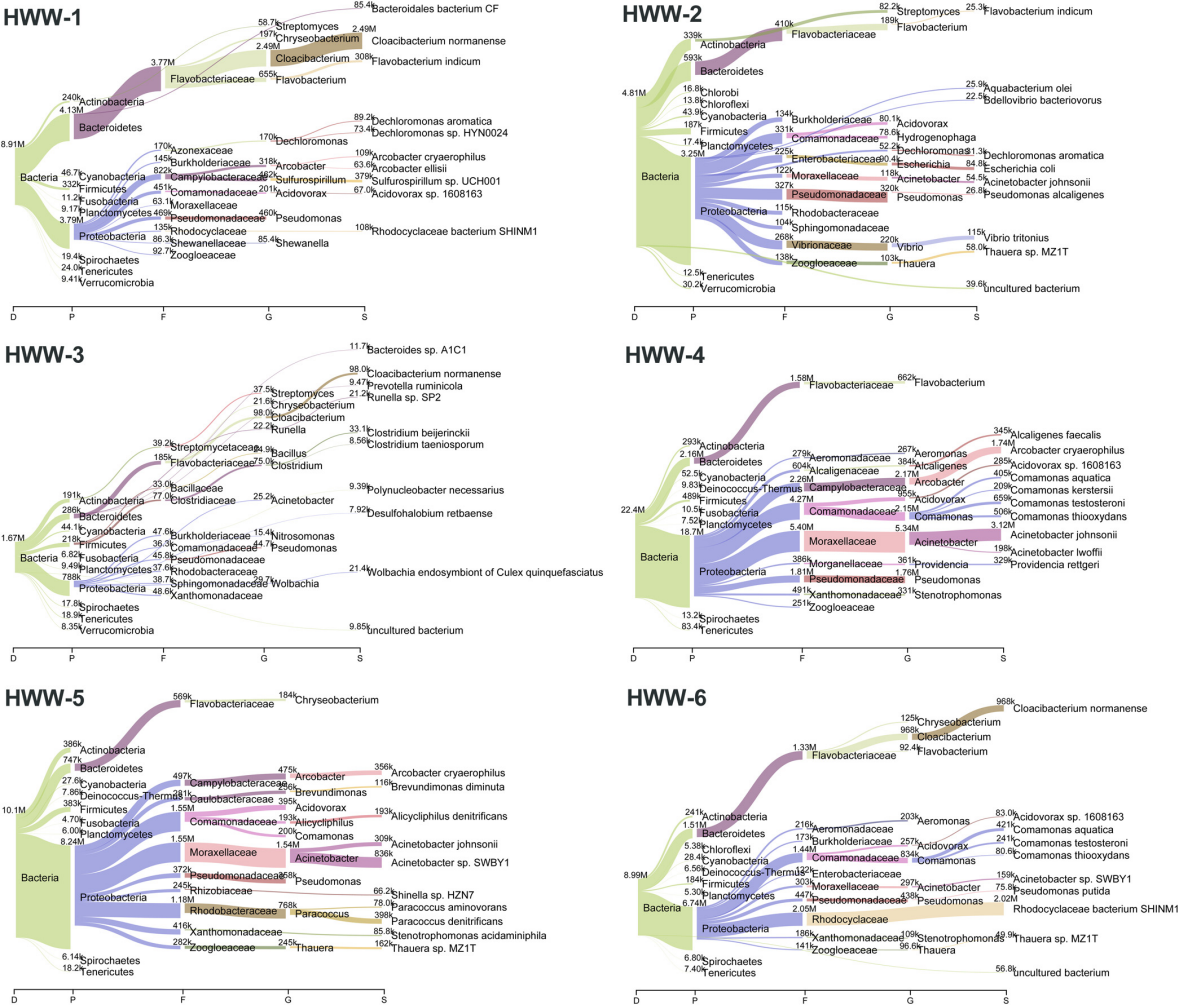
Taxonomic composition of hospital wastewater microbiomes. Sankey plots representing the abundance of various taxa in terms of reads distribution.

**FIG 3:**
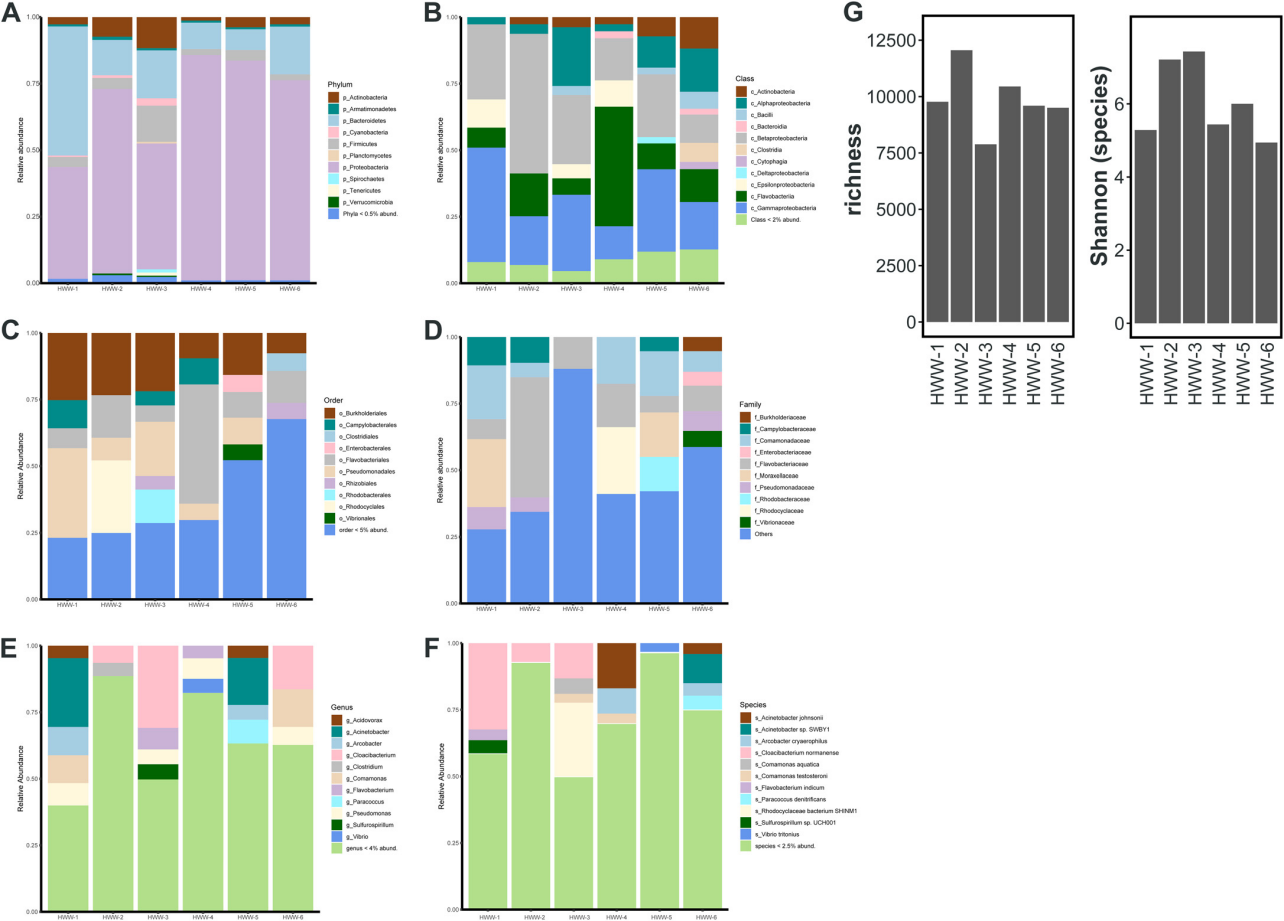
(A to F) The bacterial diversity in terms of relative abundance at various taxonomic levels in all the samples: Phyla (A), Class (B), Order (C), Family (D), Genera (E), and Species diversity (F). (G) Richness of bacterial content and alp ha diversity of microbiome in all the samples.

The HWW microbiome is highly complex, with 16 phyla, 39 classes, 108 orders, 247 families, 1,071 genera, and 7,802 species identified. *Proteobacteria* and *Bacteroidetes* were the most abundant phyla in all HWW samples (Fig. 2). *Proteobacteria* constituted approximately 80% of the bacterial population in HWW-2 and HWW-4 to HWW-6 but just half the population was formed in HWW-3 (Fig. 3A to F). HWW-1 had a distinct microbiome from the rest of the samples, with an approximately equal abundance of *Bacteroidetes* (48%) and *Proteobacteria* (≈44%). In all the other samples, *Bacteroidetes* were comparatively low (8% to 17%). Wastewater bacteria *Cloacibacterium* and *Flavobacterium* and environmental bacteria Pseudomonas were the predominant genera in HWW-1 to HWW-3, whereas Acinetobacter was dominant in HWW-4 to HWW-6. The Shannon diversity index showed similar microbiome diversity in all the samples (Fig. 3G).

### Prevalence of ARGs of urgent clinical concern in Indian HWW resistome.

**FIG 4:**
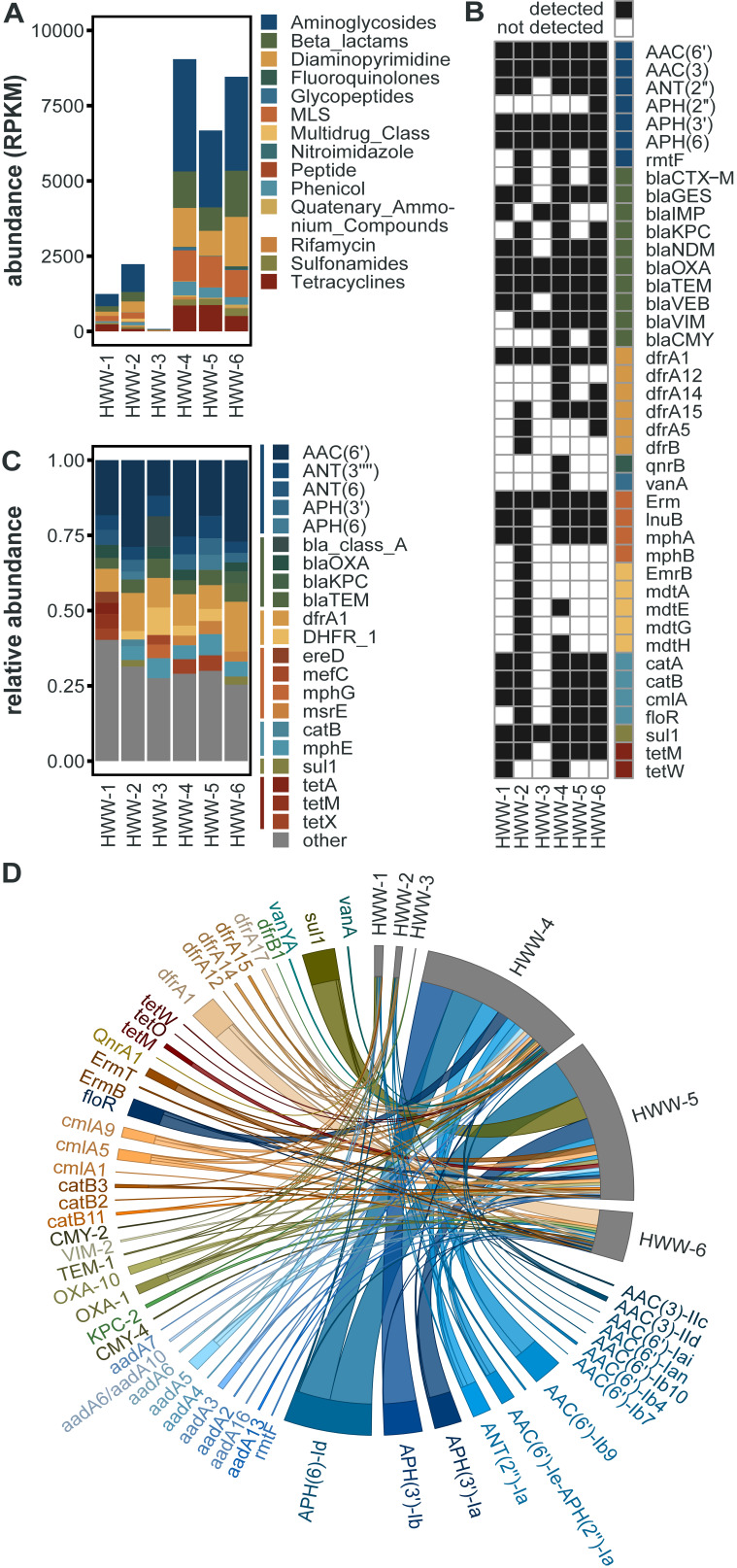
Identification of ARGs of clinical concern. (A) Abundance of identified ARG markers, quantified as reads per kilobase million (RPKM) and grouped by antibiotic drug target. (B) Presence-absence heatmap of select ARGs of clinical concern. Colored box indicates each ARG’s corresponding antibiotic drug target, colored the same as in Fig. 4A. (C) The relative abundances of the top 10 most abundant ARGs in each sample. ARGs are grouped by antibiotic drug target (thin bar), colored the same as in Fig. 4A. The relative abundances of all other ARGs not in the sample’s top 10 ARGs are grouped as “other.” (D) Abundance of ARGs of clinical concern, quantified as fragments per kilobase million (FPKM) identified through the assembly based method.

**FIG 5:**
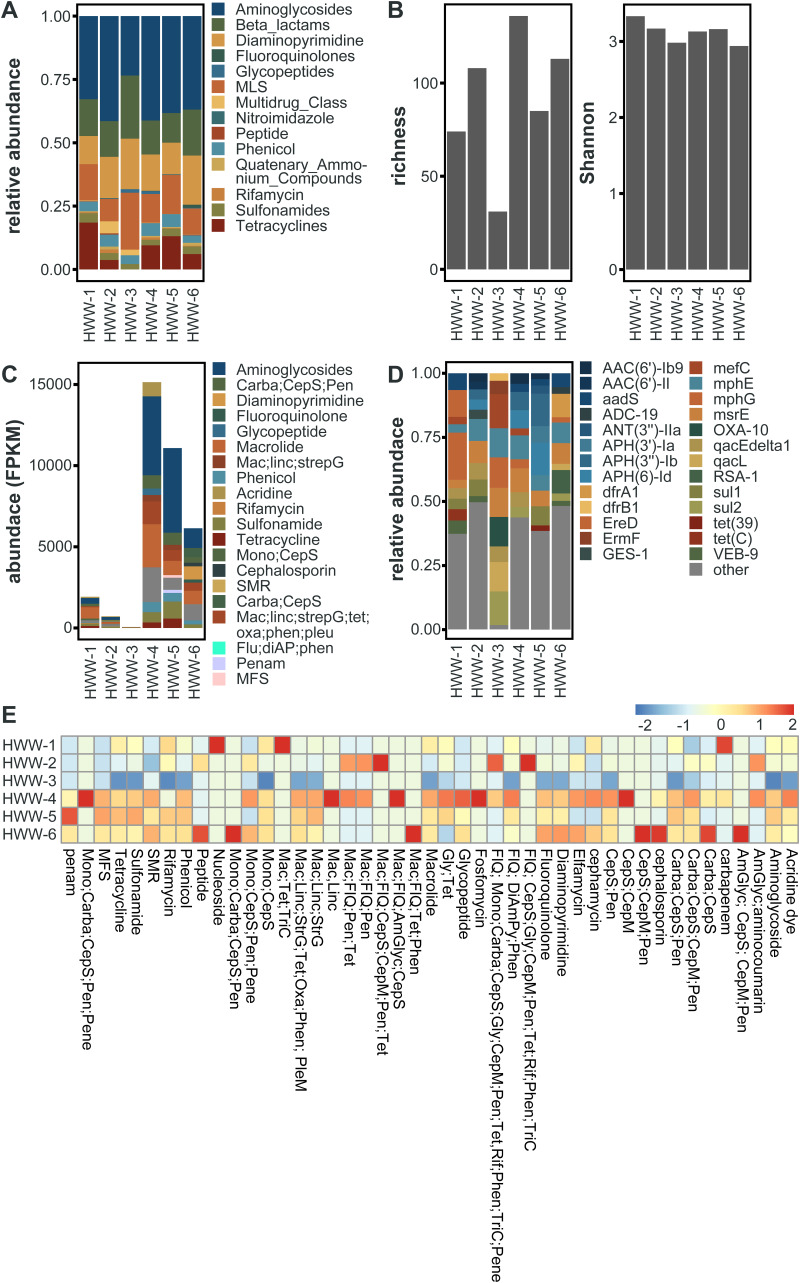
Resistome identification. (A) The relative abundance of ARGs to specific antibiotic drug classes identified by ShortBRED. (B) Richness and Shannon diversity of HWW sample ARGs identified by ShortBRED. (C) Abundance of top 10 drug classes conferring resistance across all the six samples identified by RGI. (D) Relative abundance of the top 10 ARGs in all the HWW samples, identified by RGI. (E) The heatmap of log-transformed abundance of resistance drug classes (AmGlyc, aminoglycoside; CepS, cephalosporin; CepM, cephamycin; Pen, penam; Carba, carbapenem; FlQ, fluoroquinolone; Tet, tetracycline; Rif, rifamycin; TriC, triclosan; DiAmPy, diaminopyrimidine; Phen, phenicol; Gly, glycopeptide; Mono, monobactam; Mac, macrolide; Linc, lincosamide; StrG, streptogramin; Oxa, oxazolidinone; SMR, small multidrug resistance, MFS, Major facilitator superfamily; Pene, penem).

In total, we identified 183 unique AMR determinants using ShortBRED (25). HWW-1 to HWW-3 had lower ARG abundances, quantified as reads per kilobase million (RPKM), compared to HWW-4 to HWW-6 (Fig. 4A). However, the relative abundance of ARGs to specific antibiotic drug classes was comparable across sampling sites (Fig. 5A). We detected the lowest abundance of ARGs in HWW-3, and this is also reflected in as a lower ARG richness, but the Shannon diversity of all HWW samples was comparable (Fig. 5B).

The WHO and other literature have identified several ARGs as being of urgent clinical concern because they have been associated with antibiotic treatment failure in hospitals and/or are widespread on MGEs (26). Many of these were identified in all 6 HWW samples, namely: the aminoglycoside-modifying enzymes *aac*(*6′*), *aac*(*3*), *aph*(*3′*), and *aph*(*6*), the carbapenemase *bla*_OXA_, the beta-lactamase *bla*_TEM_, the trimethoprim resistance gene *dfrA1*, the macrolide-lincosamide-streptogramin (MLS) resistance gene *Erm*, and the sulfonamide resistance gene *sul1* (Fig. 4B). Further, most of these genes are also among the top 10 most abundant ARGs in each sample (Fig. 4C), indicating that they are not only present but highly prevalent in Indian HWW, and by extension in the hospitals themselves. In addition, numerous other clinically important ARGs were identified in a subset of HWW samples (Fig. 4B), highlighting the severity of AMR propagating in the Indian HWW environment.

Other ARGs of rising concern detected include the tetracycline-inactivating enzyme *tet*(X), which has been previously identified in one isolate from India (27, 28); however, its presence in five of six HWW samples suggests it may be more prevalent than previously thought. We also detected mobile colistin resistance genes *mcr-3* and *mcr-5* in HWW-6, which represents the first time *mcr-5* was identified in Indian hospital wastewater (29).

To study ARG variants and genetic contexts, we assembled metagenomic reads and identified ARGs using the contig-based tool, RGI (Resistance Gene Identifier) (30). Although homologous ARGs belong to the same gene family, different variants exhibit substantially different risks in terms of host range, mobility potential, and ecological distribution (26). ARG abundances and compositions were consistent among this approach and ShortBRED (Fig. 5C). RGI allowed for the accurate detection of several clinically relevant high-risk ARG variants. Out of 212 unique ARG variants encompassing 43 major antibiotic classes, several were clinically concerning ARGs (53/212), often associated with MDR infections in humans (Fig. 4D). Most of these high-risk ARGs were more prevalent in HWW-4 to HWW-6, as expected owing to high ARG richness in these samples (Fig. 5C). Aminoglycoside resistance (18% to 47%) and macrolide resistance (7.9% to 36.5%) were the top two drug resistance class in all the samples, followed by carbapenemase and sulfonamide resistance. *bla*_OXA_ was the most prevalent carbapenemase across all the samples (3.6% to 11.5%) except HWW-6. In HWW-6, *bla*_RSA-1_ was the dominant carbapenemase (9.1%). Since smaller fragments of ARGs may remain undetected, we used fARGene to validate the prevalence of ARGs by reconstruction of contigs (23). Among the detected ARGs, only four beta-lactamase genes were not identified earlier by RGI, namely, *bla*_CARB-16_ and *bla*_VIM-38_ in HWW-4 and *bla*_CMY-59_ and *bla*_OXA-4_ in HWW-6.

**FIG 6:**
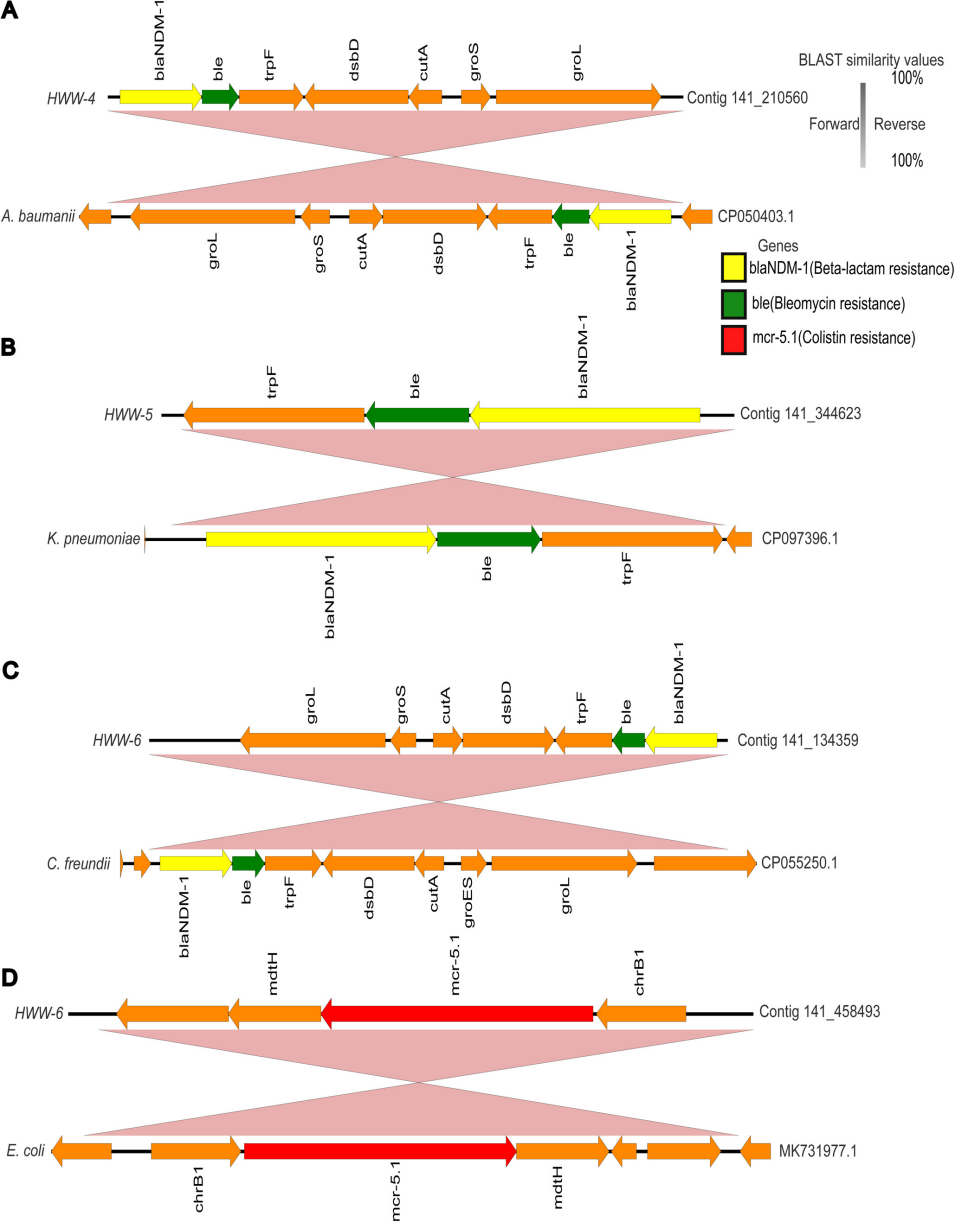
The genetic contexts of blaNDM-1 and mcr-5. (A to C) The genetic context of *bla*_NDM-1_ in HWW-4 (A), HWW-5 (B), and HWW-6 (C). (D) The genetic context of identified *mcr*-5.1 from HWW-6. The genes were annotated through Prokka and BLASTn was performed to find the best hit identity ≥99% and plotted using Easyfig v.2.1. (https://mjsull.github.io/Easyfig). The best hits are represented with accession numbers (CP050403.1, CP097396.1, CP055250.1, and MK731977.1).

Several ARGs of urgent clinical concern are endemic to India (31, 32). *bla*_NDM_, a carbapenemase conferring resistance against most β-lactam antibiotics, was first identified in a Swedish patient returning from New Delhi but in a short span of time was detected in several outbreaks around the world (33). Several studies reported multiple *bla*_NDM_ variants in J.N.M.C. hospital wastewater (HWW-1), but due to lack of surveillance at other sites of study, this is the first time that we are reporting the presence of *bla*_NDM-1_ in HWW-2, HWW-4, HWW-5, and HWW-6 (Fig. 6A to C) (34–36). *bla*_NDM-1_ often spreads through plasmid-mediated horizontal gene transfer (HGT) (37), and in this study, *bla*_NDM-1_ was predicted to be harbored by plasmid in HWW-2.

Aminoglycosides are typically not included in the first line of antibiotic treatment in many clinical scenarios, yet the high abundance of aminoglycoside resistance genes in HWW-2, HWW-4, HWW-5, and HWW-6 was surprising. As aminoglycosides are used against bacteria already resistant to beta-lactams and fluoroquinolones, the predominance of aminoglycoside resistance genes represents the presence of MDR and pan-drug-resistant bacteria in the hospital environment (38). Colistin is a last resort antibiotic for treating MDR infections, and our detection of the *mcr-5.1* gene, likely harbored on an E. coli IncX1 plasmid, is worrisome (Fig. 6D) (29).

### ARG co-occurrence and association with MGEs.

**FIG 7:**
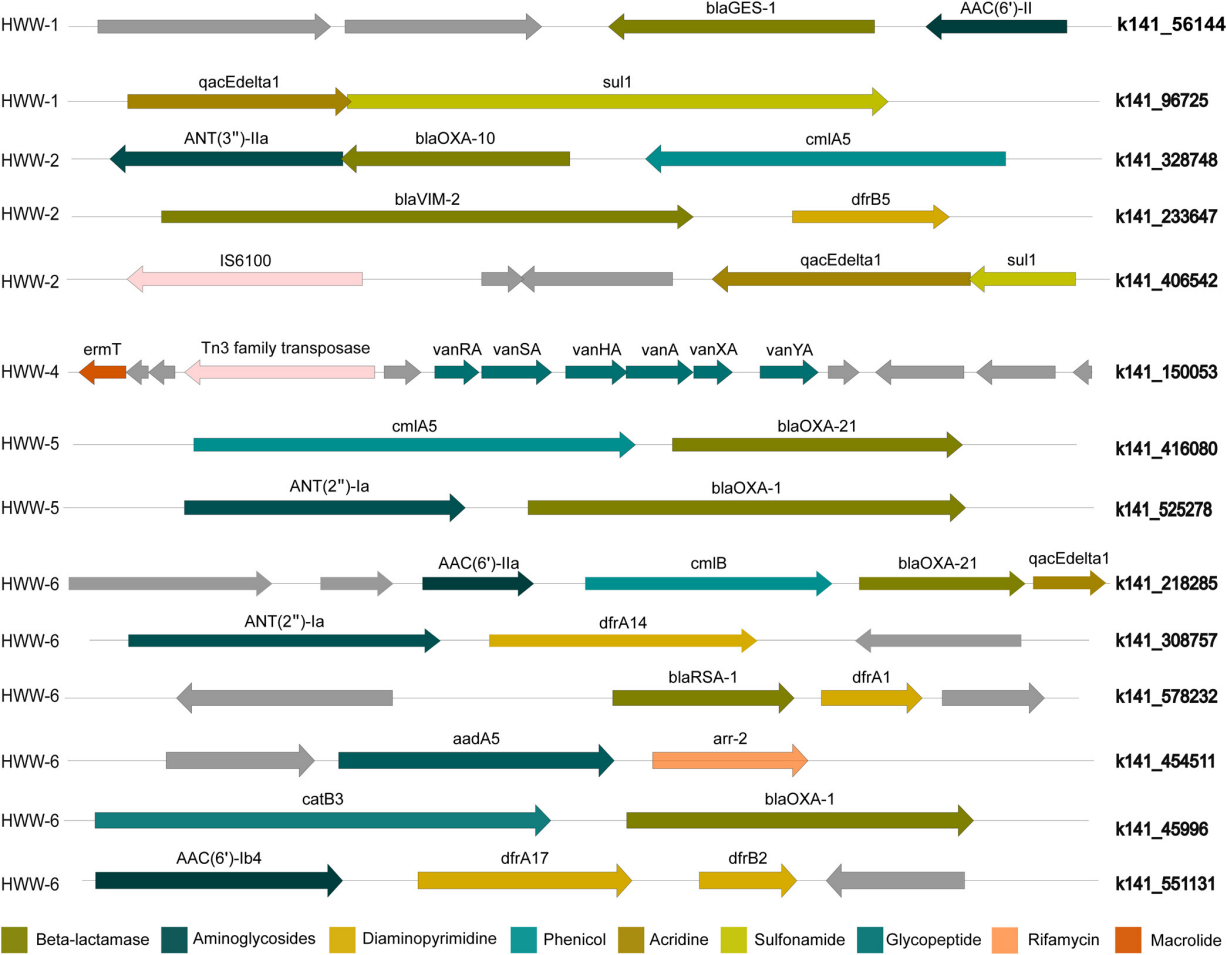
ARG co-occurrence. The genetic context of ARGs belonging to different drug classes carried on the same contig. IS element IS6100 in HWW-2 and Tn3 family transposase in HWW-4 is represented in light pink color.

Assembly-based analysis revealed the coexistence of multiple ARGs, which may cause a severe threat to the treatment of clinical infections. We identified ARGs that were present on the same contig (Fig. 7). In HWW-1, five pairs of coexisting ARGs were identified in which three pairs had resistance toward the same drug class. Among the cooccurring resistance genes belonging to different drug classes, *bla*_GES-1_, a carbapenemase, was associated with *AAC*(*6’*)-*II*, an aminoglycosidase and *qacEdelta1* (acridine dye resistance) with *sul1* (sulfonamide resistance gene). The resistance markers belonging to three different drug classes, *ANT*(*3″*)-*IIa*, *bla*_OXA-10_, and *cmlA5*, were encoded by the same contig in HWW-2, whereas in HWW-4 one contig carried seven different glycopeptide resistance genes along with one macrolide resistance gene. *bla*_NDM-1_ was coassociated with BRP(MBL) in HWW-4, HWW-5, and HWW-6 (Fig. 6A to C). In HWW-6, *bla*_OXA-21_ coexisted with *cmlB*, *AAC*(*6’*)*-IIa*, and *qacEdeltaI*.

### Co-occurrence of ARGs with plasmids and bacterial taxa.

Plasmids play a key role in bacterial ecology and evolution, particularly in regard to the spread of ARGs (39). Therefore, we next sought to identify ARGs associated with plasmids. ARGs in all the HWW samples were predominantly mediated by plasmids, including several important carbapenemases, such as *bla_OXA_* variants, *bla*_NDM-1_, *bla*_VIM_ variants, and *bla*_IMP_ variants. In HWW-5 and HWW-6, a significant proportion of ARGs was carried by plasmids (78% and 72% in HWW-5 and HWW-6, respectively).

Exploring the bacterial host of an ARG is a crucial question to be addressed for understanding their emergence and diversification (40). Hence, we identified the association of ARGs with bacterial taxa. As expected, based on their predominance in the composition of the HWW microbiome, ARGs were more associated with *Proteobacteria* with an average content of 83.8% (range, 77.9% to 91.9%) followed by *Bacteroidetes*. The ARGs were mostly carried by the genus Pseudomonas, and P. aeruginosa was the predominant species in all samples except HWW-4 where the genus Acinetobacter and species A. baumannii were most prominent. Both P. aeruginosa and *A. baumanii* ESKAPE pathogens often cause severe nosocomial infections leading to high mortalities (41, 42). We detected multiple carbapenemase genes such as *bla*_NDM_, *bla*_OXA_, *bla*_DIM_, *bla_I_*_MP_, *bla*_VIM_, and *bla*_GES_ carried by these species. The taxonomic identification of contigs carrying *bla*_NDM-1_ showed that it was carried by P. aeruginosa in HWW-2, K. pneumoniae in HWW-4 and HWW-6, and *A. baumanii* in HWW-5. Colistin resistance gene *mcr-5.1* in HWW-6 was detected in E. coli.

**FIG 8:**
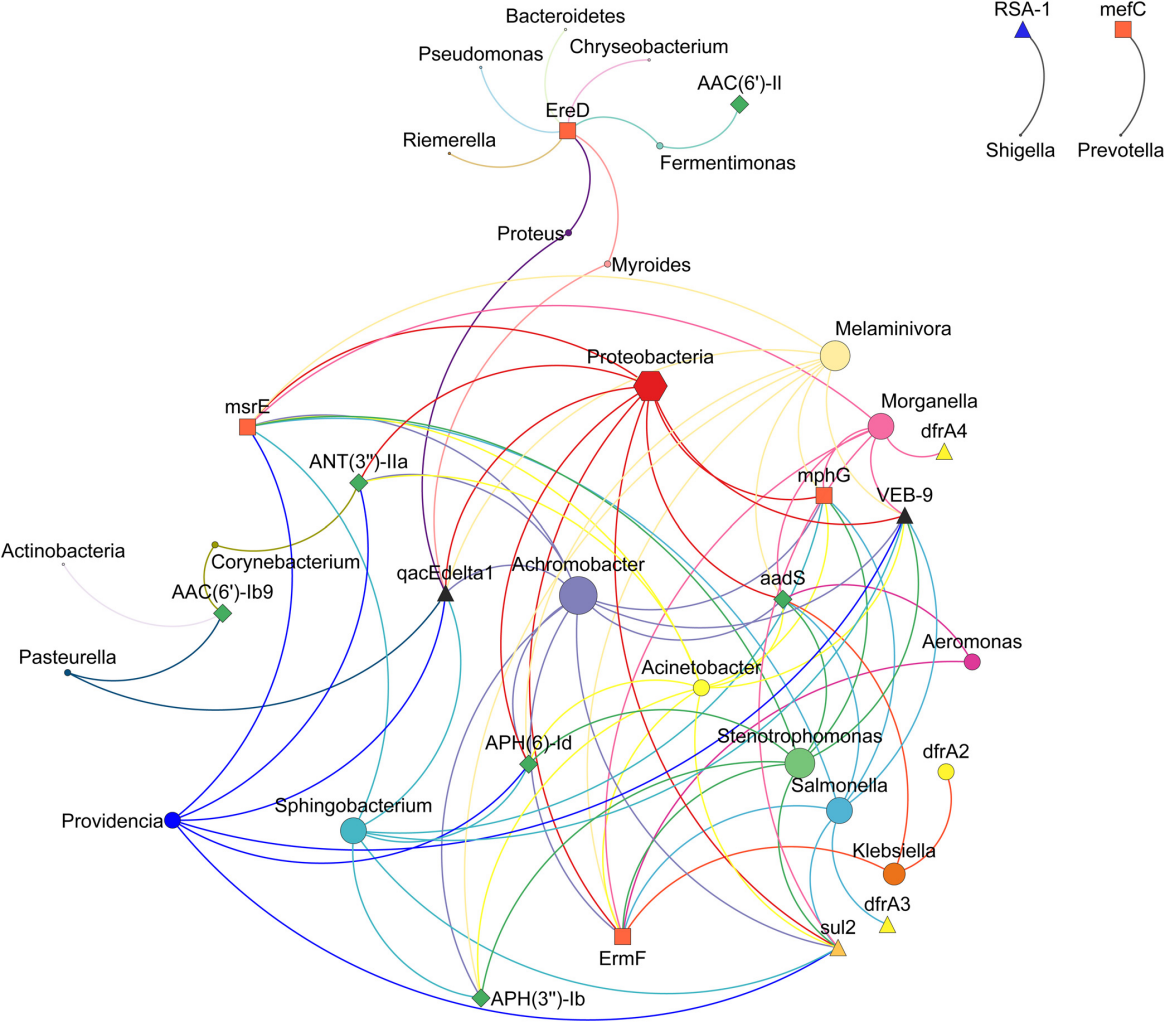
ARG-taxonomy cooccurrence network. The network analysis of cooccurrence of top 10 ARG subtypes in all the samples. The connection pattern represents the Spearman’s correlation with 0.8 cutoff value (*P* value). The different sizes of nodes are indicative of the number of connections.

Further, we explored the cooccurrence patterns between the abundance of the top 10 ARGs across all the samples and the associated bacterial abundance at phylum and genus levels. There was a significant Spearman’s rank correlation (Spearman’s ρ = 0.81~1; *P* < 0.05) between microbial diversity and ARG diversity (Fig. 8). We used network analysis to explore cooccurrence patterns between ARG subtypes and bacterial taxa. Several studies have hypothesized that nonrandom cooccurrence patterns between ARGs and microbial taxa are indicators for possible host information for ARGs if ARGs and coexisting microbial taxa have significantly similar abundance trends (Spearman’s ρ > 0.8; *P* < 0.05) (43, 44). A total of 20 genera and 3 phyla *Proteobacteria*, *Bacteroidetes*, and *Actinobacteria* were identified as possible hosts of ARGs. *Proteobacteria* was the host of aminoglycoside resistance genes [*aadS*, *ANT*(*3′’*)*-IIa*, and *APH*(*6*)*-Id*], macrolide resistance genes (*ErmF*, *mphG*, and *msrE*), sulfonamide resistance gene (*sul2*), beta-lactamase (*bla*_VEB-9_), and *qacEdelta1*.

### Virulence factor distribution.

Virulence factors are the agents of ecological connectivity that play an important role in the delocalization of AMR genes across niches through the formation of biofilms and increased infectivity. Twitching motility proteins mainly belonging to type IV pili and the flagellar proteins were prevalent in most HWW samples (Table 3). Bacteria, especially Pseudomonas spp., with twitching motility proteins have greater cytotoxicity toward the epithelial cells, enhanced transmission to other organs, and higher virulence. The flagellar proteins are responsible for adhesion, biofilm formation, and modulation of the immune system of eukaryotic cells (45). General secretion pathway proteins (gspC, gspF, gspG, gspH, gspI, gspK, and gspM) were most abundant in HWW-2 followed by type IV pili and twitching motility proteins (pilG, pilH, and pilI). In all the HWW samples, most virulence factors were associated with Pseudomonas except in HWW-2. In HWW-2, Escherichia carried most of the virulence factors. HWW-2 represented a distribution of unique VFs compared to other samples. Most of them were E. coli-specific factors like dispersin, type III secretion system effectors, general secretion pathway proteins, and E. coli common pilus chaperones. The presence of alginate biosynthesis, alginate regulation, and alginate biosynthesis virulence factors enhances the capability of bacteria to produce biofilm. The biofilm is highly proficient in transferring AMR and has an innate tolerance toward antibiotics (46). The general secretion pathway proteins have been identified as a major target for the treatment of infections and to curb the growing antibiotic resistance (47).

**TABLE 3:** Virulence factors identified through ABRicate v1.0.1 using VFDB^*a*^

Sequence	Gene	Product
HWW-1		
k141_344139	pilT	(pilT) twitching motility protein PilT [Type IV pili (VF0082)] [Pseudomonas aeruginosa PAO1]
k141_47463	pilT	(pilT) twitching motility protein PilT [Type IV pili (VF0082)] [Pseudomonas aeruginosa PAO1]
k141_47463	pilU	(pilU) twitching motility protein PilU [Type IV pili (VF0082)] [Pseudomonas aeruginosa PAO1]
k141_359157	pilG	(pilG) twitching motility protein PilG [Type IV pili (VF0082)] [Pseudomonas aeruginosa PAO1]
k141_359157	pilH	(pilH) twitching motility protein PilH [Type IV pili (VF0082)] [Pseudomonas aeruginosa PAO1]
k141_359157	pilI	(pilI) twitching motility protein PilI [Type IV pili (VF0082)] [Pseudomonas aeruginosa PAO1]
k141_359157	pilJ	(pilJ) twitching motility protein PilJ [Type IV pili (VF0082)] [Pseudomonas aeruginosa PAO1]
k141_221870	algU	(algU) alginate biosynthesis protein AlgZ/FimS [Alginate (VF0091)] [Pseudomonas aeruginosa PAO1]
k141_250443	flgC	(flgC) flagellar basal-body rod protein FlgC [Flagella (VF0273)] [Pseudomonas aeruginosa PAO1]
k141_272187	flgC	(flgC) flagellar basal-body rod protein FlgC [Flagella (VF0273)] [Pseudomonas aeruginosa PAO1]
k141_211659	flgH	(flgH) flagellar L-ring protein precursor FlgH [Flagella (VF0273)] [Pseudomonas aeruginosa PAO1]
k141_283651	flgI	(flgI) flagellar P-ring protein precursor FlgI [Flagella (VF0273)] [Pseudomonas aeruginosa PAO1]
k141_316175	flgI	(flgI) flagellar P-ring protein precursor FlgI [Flagella (VF0273)] [Pseudomonas aeruginosa PAO1]
k141_132209	fliE	(fliE) flagellar hook-basal body complex protein FliE [Flagella (VF0273)] [Pseudomonas aeruginosa PAO1]
k141_446999	fliG	(fliG) flagellar motor switch protein G [Flagella (VF0273)] [Pseudomonas aeruginosa PAO1]
k141_61688	fliI	(fliI) flagellum-specific ATP synthase FliI [Flagella (VF0273)] [Pseudomonas aeruginosa PAO1]
k141_346249	fliM	(fliM) flagellar motor switch protein FliM [Flagella (VF0273)] [Pseudomonas aeruginosa PAO1]
k141_346249	fliN	(fliN) flagellar motor switch protein FliN [Flagella (VF0273)] [Pseudomonas aeruginosa PAO1]
k141_483093	fliP	(fliP) flagellar biosynthetic protein FliP [Flagella (VF0273)] [Pseudomonas aeruginosa PAO1]
k141_483093	fliQ	(fliQ) flagellar biosynthetic protein FliQ [Flagella (VF0273)] [Pseudomonas aeruginosa PAO1]
k141_24580	flhA	(flhA) flagellar biosynthesis protein FlhA [Flagella (VF0273)] [Pseudomonas aeruginosa PAO1]
k141_160638	fleN	(fleN) flagellar synthesis regulator FleN [Flagella (VF0273)] [Pseudomonas aeruginosa PAO1]
k141_153162	xcpT	(xcpT) general secretion pathway protein G [xcp secretion system (VF0084)] [Pseudomonas aeruginosa PAO1]
k141_153162	xcpS	(xcpS) general secretion pathway protein F [xcp secretion system (VF0084)] [Pseudomonas aeruginosa PAO1]
k141_153162	xcpR	(xcpR) general secretion pathway protein E [xcp secretion system (VF0084)] [Pseudomonas aeruginosa PAO1]
k141_250831	algW	(algW) AlgW protein [Alginate regulation (CVF523)] [Pseudomonas aeruginosa PAO1]
k141_438174	algR	(algR) alginate biosynthesis regulatory protein AlgR [Alginate (VF0091)] [Pseudomonas aeruginosa PAO1]
k141_440675	algR	(algR) alginate biosynthesis regulatory protein AlgR [Alginate (VF0091)] [Pseudomonas aeruginosa PAO1]
k141_219894	algC	(algC) phosphomannomutase AlgC [Alginate biosynthesis (CVF522)] [Pseudomonas aeruginosa PAO1]
HWW-2		
k141_340702	aap/aspU	(aap/aspU) Dispersin [Dispersin (VF0215)] [Escherichia coli O44:H18 042]
k141_275495	aggB	(aggB) fimbrial minor subunit [AAFs (VF0214)] [Escherichia coli 17-2]
k141_203453	csgD	(csgD) DNA-binding transcriptional regulator CsgD [curli fibers/thin aggregative fimbriae (AGF) (AI094)] [Salmonella enterica subsp. enterica serovar Typhimurium str. LT2]
k141_203453	csgF	(csgF) curli production assembly/transport protein CsgF [Agf (VF0103)] [Salmonella enterica subsp. enterica serovar Typhimurium str. LT2]
k141_200776	espR4	(espR4) Type III secretion system effector espR4 [LEE encoded T3SS (SS020)] [Escherichia coli O157:H7 str. EDL933]
k141_85666	espY1	(espY1) Type III secretion system effector EspY1 [LEE encoded T3SS (SS020)] [Escherichia coli O157:H7 str. EDL933]
k141_146443	fepB	(fepB) ferrienterobactin ABC transporter periplasmic binding protein [Enterobactin (VF0228)] [Escherichia coli CFT073]
k141_441654	fepD	(fepD) ferrienterobactin ABC transporter permease [Enterobactin (VF0228)] [Escherichia coli CFT073]
k141_63063	fimI	(fimI) Fimbrin-like protein fimI precursor [Type 1 fimbriae (VF0221)] [Escherichia coli CFT073]
k141_319634	fliQ	(fliQ) flagellar biosynthetic protein FliQ [Flagella (VF0273)] [Pseudomonas aeruginosa PAO1]
k141_272515	gspC	(gspC) general secretion pathway protein C [T2SS (VF0333)] [Shigella dysenteriae Sd197]
k141_181603	gspF	(gspF) general secretion pathway protein F [T2SS (VF0333)] [Shigella dysenteriae Sd197]
k141_181603	gspG	(gspG) general secretion pathway protein G [T2SS (VF0333)] [Shigella dysenteriae Sd197]
k141_234431	gspH	(gspH) general secretion pathway protein H [T2SS (VF0333)] [Shigella dysenteriae Sd197]
k141_166675	gspI	(gspI) general secretion pathway protein I [T2SS (VF0333)] [Shigella dysenteriae Sd197]
k141_203361	gspK	(gspK) general secretion pathway protein K [T2SS (VF0333)] [Shigella dysenteriae Sd197]
k141_799	gspM	(gspM) general secretion pathway protein M [T2SS (VF0333)] [Shigella dysenteriae Sd197]
k141_255827	hcp1	(hcp1) type VI secretion system substrate Hcp1 [HSI-I (VF0334)] [Pseudomonas aeruginosa PAO1]
k141_295605	kpsM	(kpsM) KpsM [K1 capsule (VF0239)] [Escherichia coli O18:K1:H7 str. RS218]
k141_24329	pilG	(pilG) twitching motility protein PilG [Type IV pili (VF0082)] [Pseudomonas aeruginosa PAO1]
k141_69666	pilG	(pilG) twitching motility protein PilG [Type IV pili (VF0082)] [Pseudomonas aeruginosa PAO1]
k141_356469	pilH	(pilH) twitching motility protein PilH [Type IV pili (VF0082)] [Pseudomonas aeruginosa PAO1]
k141_48103	pilI	(pilI) twitching motility protein PilI [Type IV pili (VF0082)] [Pseudomonas aeruginosa PAO1]
k141_44828	shuX	(shuX) shu locus protein ShuX [Shu (VF0256)] [Shigella dysenteriae Sd197]
k141_466702	yagV/ecpE	(yagV/ecpE) E. coli common pilus chaperone EcpE [ECP (VF0404)] [Escherichia coli O157:H7 str. EDL933]
k141_299262	yagX/ecpC	(yagX/ecpC) E. coli common pilus usher EcpC [ECP (VF0404)] [Escherichia coli O157:H7 str. EDL933]
k141_299262	yagY/ecpB	(yagY/ecpB) E. coli common pilus chaperone EcpB [ECP (VF0404)] [Escherichia coli O157:H7 str. EDL933]
k141_439431	yagZ/ecpA	(yagZ/ecpA) E. coli common pilus structural subunit EcpA [ECP (VF0404)] [Escherichia coli O157:H7 str. EDL933]
k141_439431	ykgK/ecpR	(ykgK/ecpR) regulator protein EcpR [ECP (VF0404)] [Escherichia coli O157:H7 str. EDL933]
HWW-4		
k141_125532	acpXL	(acpXL) acyl carrier protein [LPS (CVF383)] [Brucella melitensis bv. 1 str. 16M]
k141_142010	acpXL	(acpXL) acyl carrier protein [LPS (CVF383)] [Brucella melitensis bv. 1 str. 16M]
k141_298551	acpXL	(acpXL) acyl carrier protein [LPS (CVF383)] [Brucella melitensis bv. 1 str. 16M]
k141_453859	acpXL	(acpXL) acyl carrier protein [LPS (CVF383)] [Brucella melitensis bv. 1 str. 16M]
k141_553919	alg8	(alg8) alginate-c5-mannuronan-epimerase AlgG [Alginate (VF0091)] [Pseudomonas aeruginosa PAO1]
k141_446658	algA	(algA) phosphomannose isomerase / guanosine 5′-diphospho-D-mannose pyrophosphorylase [Alginate (VF0091)] [Pseudomonas aeruginosa PAO1]
k141_200944	algB	(algB) two-component response regulator AlgB [Alginate (VF0091)] [Pseudomonas aeruginosa PAO1]
k141_105172	algC	(algC) phosphomannomutase AlgC [Alginate biosynthesis (CVF522)] [Pseudomonas aeruginosa PAO1]
k141_485400	algD	(algD) GDP-mannose 6-dehydrogenase AlgD [Alginate (VF0091)] [Pseudomonas aeruginosa PAO1]
k141_12932	algI	(algI) alginate o-acetyltransferase AlgI [Alginate (VF0091)] [Pseudomonas aeruginosa PAO1]
k141_50607	algR	(algR) alginate biosynthesis regulatory protein AlgR [Alginate (VF0091)] [Pseudomonas aeruginosa PAO1]
k141_185855	algU	(algU) alginate biosynthesis protein AlgZ/FimS [Alginate (VF0091)] [Pseudomonas aeruginosa PAO1]
k141_459309	algU	(algU) alginate biosynthesis protein AlgZ/FimS [Alginate (VF0091)] [Pseudomonas aeruginosa PAO1]
k141_507216	algU	(algU) alginate biosynthesis protein AlgZ/FimS [Alginate (VF0091)] [Pseudomonas aeruginosa PAO1]
k141_568819	algU	(algU) alginate biosynthesis protein AlgZ/FimS [Alginate (VF0091)] [Pseudomonas aeruginosa PAO1]
k141_589694	algU	(algU) alginate biosynthesis protein AlgZ/FimS [Alginate (VF0091)] [Pseudomonas aeruginosa PAO1]
k141_415468	fimF	(fimF) FimF protein precursor [Type 1 fimbriae (VF0221)] [Escherichia coli CFT073]
k141_357125	fleN	(fleN) flagellar synthesis regulator FleN [Flagella (VF0273)] [Pseudomonas aeruginosa PAO1]
k141_340898	fleQ	(fleQ) transcriptional regulator FleQ [Flagella (VF0273)] [Pseudomonas aeruginosa PAO1]
k141_113633	flgC	(flgC) flagellar basal-body rod protein FlgC [Flagella (VF0273)] [Pseudomonas aeruginosa PAO1]
k141_253387	flgC	(flgC) flagellar basal-body rod protein FlgC [Flagella (VF0273)] [Pseudomonas aeruginosa PAO1]
k141_428864	flgC	(flgC) flagellar basal-body rod protein FlgC [Flagella (VF0273)] [Pseudomonas aeruginosa PAO1]
k141_343807	flgG	(flgG) flagellar basal-body rod protein FlgG [Flagella (VF0273)] [Pseudomonas aeruginosa PAO1]
k141_346193	flgG	(flgG) flagellar basal-body rod protein FlgG [Flagella (VF0273)] [Pseudomonas aeruginosa PAO1]
k141_274809	flgH	(flgH) flagellar L-ring protein precursor FlgH [Flagella (VF0273)] [Pseudomonas aeruginosa PAO1]
k141_591610	flgH	(flgH) flagellar L-ring protein precursor FlgH [Flagella (VF0273)] [Pseudomonas aeruginosa PAO1]
k141_274809	flgI	(flgI) flagellar P-ring protein precursor FlgI [Flagella (VF0273)] [Pseudomonas aeruginosa PAO1]
k141_318595	flhA	(flhA) flagellar biosynthesis protein FlhA [Flagella (VF0273)] [Pseudomonas aeruginosa PAO1]
k141_141825	fliA	(fliA) flagellar biosynthesis sigma factor FliA [Deoxyhexose linking sugar 209 Da capping structure (AI138)] [Pseudomonas aeruginosa PAO1]
k141_264415	fliA	(fliA) flagellar biosynthesis sigma factor FliA [Deoxyhexose linking sugar 209 Da capping structure (AI138)] [Pseudomonas aeruginosa PAO1]
k141_397047	fliE	(fliE) flagellar hook-basal body complex protein FliE [Flagella (VF0273)] [Pseudomonas aeruginosa PAO1]
k141_106581	fliG	(fliG) flagellar motor switch protein G [Flagella (VF0273)] [Pseudomonas aeruginosa PAO1]
k141_3328	fliG	(fliG) flagellar motor switch protein G [Flagella (VF0273)] [Pseudomonas aeruginosa PAO1]
k141_266069	fliI	(fliI) flagellum-specific ATP synthase FliI [Flagella (VF0273)] [Pseudomonas aeruginosa PAO1]
k141_79328	fliI	(fliI) flagellum-specific ATP synthase FliI [Flagella (VF0273)] [Pseudomonas aeruginosa PAO1]
k141_207084	fliM	(fliM) flagellar motor switch protein FliM [Flagella (VF0273)] [Pseudomonas aeruginosa PAO1]
k141_219044	fliM	(fliM) flagellar motor switch protein FliM [Flagella (VF0273)] [Pseudomonas aeruginosa PAO1]
k141_433887	fliM	(fliM) flagellar motor switch protein FliM [Flagella (VF0273)] [Pseudomonas aeruginosa PAO1]
k141_443952	fliM	(fliM) flagellar motor switch protein FliM [Flagella (VF0273)] [Pseudomonas aeruginosa PAO1]
k141_180332	fliN	(fliN) flagellar motor switch protein FliN [Flagella (VF0273)] [Pseudomonas aeruginosa PAO1]
k141_219044	fliN	(fliN) flagellar motor switch protein FliN [Flagella (VF0273)] [Pseudomonas aeruginosa PAO1]
k141_186333	fliP	(fliP) flagellar biosynthetic protein FliP [Flagella (VF0273)] [Pseudomonas aeruginosa PAO1]
k141_433887	fliP	(fliP) flagellar biosynthetic protein FliP [Flagella (VF0273)] [Pseudomonas aeruginosa PAO1]
k141_72397	fliP	(fliP) flagellar biosynthetic protein FliP [Flagella (VF0273)] [Pseudomonas aeruginosa PAO1]
k141_88240	fliP	(fliP) flagellar biosynthetic protein FliP [Flagella (VF0273)] [Pseudomonas aeruginosa PAO1]
k141_396457	fliQ	(fliQ) flagellar biosynthetic protein FliQ [Flagella (VF0273)] [Pseudomonas aeruginosa PAO1]
k141_40634	fliQ	(fliQ) flagellar biosynthetic protein FliQ [Flagella (VF0273)] [Pseudomonas aeruginosa PAO1]
k141_127626	mbtH-like	(mbtH-like) MbtH-like protein from the pyoverdine cluster [pyoverdine (IA001)] [Pseudomonas aeruginosa PAO1]
k141_567320	motA	(motA) flagellar motor protein [Deoxyhexose linking sugar 209 Da capping structure (AI138)] [Pseudomonas aeruginosa PAO1]
k141_430957	motC	(motC) flagellar motor protein [Deoxyhexose linking sugar 209 Da capping structure (AI138)] [Pseudomonas aeruginosa PAO1]
k141_14328	pilG	(pilG) twitching motility protein PilG [Type IV pili (VF0082)] [Pseudomonas aeruginosa PAO1]
k141_16339	pilG	(pilG) twitching motility protein PilG [Type IV pili (VF0082)] [Pseudomonas aeruginosa PAO1]
k141_245324	pilG	(pilG) twitching motility protein PilG [Type IV pili (VF0082)] [Pseudomonas aeruginosa PAO1]
k141_545762	pilG	(pilG) twitching motility protein PilG [Type IV pili (VF0082)] [Pseudomonas aeruginosa PAO1]
k141_55210	pilG	(pilG) twitching motility protein PilG [Type IV pili (VF0082)] [Pseudomonas aeruginosa PAO1]
k141_589547	pilG	(pilG) twitching motility protein PilG [Type IV pili (VF0082)] [Pseudomonas aeruginosa PAO1]
k141_597392	pilG	(pilG) twitching motility protein PilG [Type IV pili (VF0082)] [Pseudomonas aeruginosa PAO1]
k141_624550	pilG	(pilG) twitching motility protein PilG [Type IV pili (VF0082)] [Pseudomonas aeruginosa PAO1]
k141_82558	pilG	(pilG) twitching motility protein PilG [Type IV pili (VF0082)] [Pseudomonas aeruginosa PAO1]
k141_119537	pilH	(pilH) twitching motility protein PilH [Type IV pili (VF0082)] [Pseudomonas aeruginosa PAO1]
k141_333795	pilH	(pilH) twitching motility protein PilH [Type IV pili (VF0082)] [Pseudomonas aeruginosa PAO1]
k141_445530	pilH	(pilH) twitching motility protein PilH [Type IV pili (VF0082)] [Pseudomonas aeruginosa PAO1]
k141_592421	pilH	(pilH) twitching motility protein PilH [Type IV pili (VF0082)] [Pseudomonas aeruginosa PAO1]
k141_597392	pilH	(pilH) twitching motility protein PilH [Type IV pili (VF0082)] [Pseudomonas aeruginosa PAO1]
k141_82558	pilH	(pilH) twitching motility protein PilH [Type IV pili (VF0082)] [Pseudomonas aeruginosa PAO1]
k141_314471	pilM	(pilM) type IV pilus inner membrane platform protein PilM [Type IV pili (VF0082)] [Pseudomonas aeruginosa PAO1]
k141_29595	pilO	(pilO) type IV pilus inner membrane platform protein PilO [Type IV pili (VF0082)] [Pseudomonas aeruginosa PAO1]
k141_553621	pilO	(pilO) type IV pilus inner membrane platform protein PilO [Type IV pili (VF0082)] [Pseudomonas aeruginosa PAO1]
k141_44074	pilR	(pilR) two-component response regulator PilR [Type IV pili (VF0082)] [Pseudomonas aeruginosa PAO1]
k141_322422	pilT	(pilT) twitching motility protein PilT [Type IV pili (VF0082)] [Pseudomonas aeruginosa PAO1]
k141_419027	pilT	(pilT) twitching motility protein PilT [Type IV pili (VF0082)] [Pseudomonas aeruginosa PAO1]
k141_113933	pilU	(pilU) twitching motility protein PilU [Type IV pili (VF0082)] [Pseudomonas aeruginosa PAO1]
k141_111109	waaA	(waaA) lipopolysaccharide core biosynthesis protein WaaP [LPS (VF0085)] [Pseudomonas aeruginosa PAO1]
k141_395234	waaF	(waaF) heptosyltransferase I [LPS (VF0085)] [Pseudomonas aeruginosa PAO1]
k141_382804	waaG	(waaG) B-band O-antigen polymerase [LPS (VF0085)] [Pseudomonas aeruginosa PAO1]
k141_220982	waaP	(waaP) UDP-glucose:(heptosyl) LPS alpha 13-glucosyltransferase WaaG [LPS (VF0085)] [Pseudomonas aeruginosa PAO1]
k141_60569	xcpA/pilD	(xcpA/pilD) type 4 prepilin peptidase PilD [Type IV pili (VF0082)] [Pseudomonas aeruginosa PAO1]
k141_222010	xcpT	(xcpT) general secretion pathway protein G [xcp secretion system (VF0084)] [Pseudomonas aeruginosa PAO1]
k141_366811	xcpT	(xcpT) general secretion pathway protein G [xcp secretion system (VF0084)] [Pseudomonas aeruginosa PAO1]
HWW-5		
k141_107730	acpXL	(acpXL) acyl carrier protein [LPS (CVF383)] [Brucella melitensis bv. 1 str. 16M]
k141_122640	waaG	(waaG) B-band O-antigen polymerase [LPS (VF0085)] [Pseudomonas aeruginosa PAO1]
k141_122640	waaP	(waaP) UDP-glucose:(heptosyl) LPS alpha 13-glucosyltransferase WaaG [LPS (VF0085)] [Pseudomonas aeruginosa PAO1]
k141_125756	acpXL	(acpXL) acyl carrier protein [LPS (CVF383)] [Brucella melitensis bv. 1 str. 16M]
k141_149358	fleN	(fleN) flagellar synthesis regulator FleN [Flagella (VF0273)] [Pseudomonas aeruginosa PAO1]
k141_154646	pilG	(pilG) twitching motility protein PilG [Type IV pili (VF0082)] [Pseudomonas aeruginosa PAO1]
k141_154646	pilH	(pilH) twitching motility protein PilH [Type IV pili (VF0082)] [Pseudomonas aeruginosa PAO1]
k141_154646	pilJ	(pilJ) twitching motility protein PilJ [Type IV pili (VF0082)] [Pseudomonas aeruginosa PAO1]
k141_154979	fliM	(fliM) flagellar motor switch protein FliM [Flagella (VF0273)] [Pseudomonas aeruginosa PAO1]
k141_161953	waaF	(waaF) heptosyltransferase I [LPS (VF0085)] [Pseudomonas aeruginosa PAO1]
k141_173408	pilH	(pilH) twitching motility protein PilH [Type IV pili (VF0082)] [Pseudomonas aeruginosa PAO1]
k141_173408	pilG	(pilG) twitching motility protein PilG [Type IV pili (VF0082)] [Pseudomonas aeruginosa PAO1]
k141_214052	pilT	(pilT) twitching motility protein PilT [Type IV pili (VF0082)] [Pseudomonas aeruginosa PAO1]
k141_236530	fliP	(fliP) flagellar biosynthetic protein FliP [Flagella (VF0273)] [Pseudomonas aeruginosa PAO1]
k141_236530	fliM	(fliM) flagellar motor switch protein FliM [Flagella (VF0273)] [Pseudomonas aeruginosa PAO1]
k141_236530	fliG	(fliG) flagellar motor switch protein G [Flagella (VF0273)] [Pseudomonas aeruginosa PAO1]
k141_237176	pilG	(pilG) twitching motility protein PilG [Type IV pili (VF0082)] [Pseudomonas aeruginosa PAO1]
k141_237176	pilH	(pilH) twitching motility protein PilH [Type IV pili (VF0082)] [Pseudomonas aeruginosa PAO1]
k141_260022	acpXL	(acpXL) acyl carrier protein [LPS (CVF383)] [Brucella melitensis bv. 1 str. 16M]
k141_275125	flgG	(flgG) flagellar basal-body rod protein FlgG [Flagella (VF0273)] [Pseudomonas aeruginosa PAO1]
k141_352540	algC	(algC) phosphomannomutase AlgC [Alginate biosynthesis (CVF522)] [Pseudomonas aeruginosa PAO1]
k141_35606	acpXL	(acpXL) acyl carrier protein [LPS (CVF383)] [Brucella melitensis bv. 1 str. 16M]
k141_360749	pilG	(pilG) twitching motility protein PilG [Type IV pili (VF0082)] [Pseudomonas aeruginosa PAO1]
k141_38661	pilT	(pilT) twitching motility protein PilT [Type IV pili (VF0082)] [Pseudomonas aeruginosa PAO1]
k141_416308	fliM	(fliM) flagellar motor switch protein FliM [Flagella (VF0273)] [Pseudomonas aeruginosa PAO1]
k141_416308	fliN	(fliN) flagellar motor switch protein FliN [Flagella (VF0273)] [Pseudomonas aeruginosa PAO1]
k141_416308	fliP	(fliP) flagellar biosynthetic protein FliP [Flagella (VF0273)] [Pseudomonas aeruginosa PAO1]
k141_424073	pilT	(pilT) twitching motility protein PilT [Type IV pili (VF0082)] [Pseudomonas aeruginosa PAO1]
k141_425540	pilU	(pilU) twitching motility protein PilU [Type IV pili (VF0082)] [Pseudomonas aeruginosa PAO1]
k141_427332	pilU	(pilU) twitching motility protein PilU [Type IV pili (VF0082)] [Pseudomonas aeruginosa PAO1]
k141_461073	pilT	(pilT) twitching motility protein PilT [Type IV pili (VF0082)] [Pseudomonas aeruginosa PAO1]
k141_461073	pilU	(pilU) twitching motility protein PilU [Type IV pili (VF0082)] [Pseudomonas aeruginosa PAO1]
k141_465653	pilG	(pilG) twitching motility protein PilG [Type IV pili (VF0082)] [Pseudomonas aeruginosa PAO1]
k141_470086	algU	(algU) alginate biosynthesis protein AlgZ/FimS [Alginate (VF0091)] [Pseudomonas aeruginosa PAO1]
k141_476116	acpXL	(acpXL) acyl carrier protein [LPS (CVF383)] [Brucella melitensis bv. 1 str. 16M]
k141_482681	xcpT	(xcpT) general secretion pathway protein G [xcp secretion system (VF0084)] [Pseudomonas aeruginosa PAO1]
k141_488285	pilT	(pilT) twitching motility protein PilT [Type IV pili (VF0082)] [Pseudomonas aeruginosa PAO1]
k141_498478	fliG	(fliG) flagellar motor switch protein G [Flagella (VF0273)] [Pseudomonas aeruginosa PAO1]
k141_562960	pilR	(pilR) two-component response regulator PilR [Type IV pili (VF0082)] [Pseudomonas aeruginosa PAO1]
k141_57587	algR	(algR) alginate biosynthesis regulatory protein AlgR [Alginate (VF0091)] [Pseudomonas aeruginosa PAO1]
k141_60943	flgI	(flgI) flagellar P-ring protein precursor FlgI [Flagella (VF0273)] [Pseudomonas aeruginosa PAO1]
k141_60943	flgG	(flgG) flagellar basal-body rod protein FlgG [Flagella (VF0273)] [Pseudomonas aeruginosa PAO1]
k141_69665	fliM	(fliM) flagellar motor switch protein FliM [Flagella (VF0273)] [Pseudomonas aeruginosa PAO1]
HWW-6		
k141_14239	pvdS	(pvdS) extracytoplasmic-function sigma-70 factor [Pyoverdine (VF0094)] [Pseudomonas aeruginosa PAO1]
k141_150252	pilG	(pilG) twitching motility protein PilG [Type IV pili (VF0082)] [Pseudomonas aeruginosa PAO1]
k141_191571	mbtH-like	(mbtH-like) MbtH-like protein from the pyoverdine cluster [pyoverdine (IA001)] [Pseudomonas aeruginosa PAO1]
k141_207228	pilT	(pilT) twitching motility protein PilT [Type IV pili (VF0082)] [Pseudomonas aeruginosa PAO1]
k141_218040	waaG	(waaG) B-band O-antigen polymerase [LPS (VF0085)] [Pseudomonas aeruginosa PAO1]
k141_218555	fliE	(fliE) flagellar hook-basal body complex protein FliE [Flagella (VF0273)] [Pseudomonas aeruginosa PAO1]
k141_232199	pilG	(pilG) twitching motility protein PilG [Type IV pili (VF0082)] [Pseudomonas aeruginosa PAO1]
k141_232199	pilH	(pilH) twitching motility protein PilH [Type IV pili (VF0082)] [Pseudomonas aeruginosa PAO1]
k141_267491	waaF	(waaF) heptosyltransferase I [LPS (VF0085)] [Pseudomonas aeruginosa PAO1]
k141_270282	algR	(algR) alginate biosynthesis regulatory protein AlgR [Alginate (VF0091)] [Pseudomonas aeruginosa PAO1]
k141_297256	hsiB1/vipA	(hsiB1/vipA) type VI secretion system tubule-forming protein VipA [HSI-I (VF0334)] [Pseudomonas aeruginosa PAO1]
k141_322579	algD	(algD) GDP-mannose 6-dehydrogenase AlgD [Alginate (VF0091)] [Pseudomonas aeruginosa PAO1]
k141_32858	fliA	(fliA) flagellar biosynthesis sigma factor FliA [Deoxyhexose linking sugar 209 Da capping structure (AI138)] [Pseudomonas aeruginosa PAO1]
k141_344288	waaP	(waaP) UDP-glucose:(heptosyl) LPS alpha 13-glucosyltransferase WaaG [LPS (VF0085)] [Pseudomonas aeruginosa PAO1]
k141_354036	hcp1	(hcp1) type VI secretion system substrate Hcp1 [HSI-I (VF0334)] [Pseudomonas aeruginosa PAO1]
k141_371808	pilG	(pilG) twitching motility protein PilG [Type IV pili (VF0082)] [Pseudomonas aeruginosa PAO1]
k141_378363	algR	(algR) alginate biosynthesis regulatory protein AlgR [Alginate (VF0091)] [Pseudomonas aeruginosa PAO1]
k141_37950	pilH	(pilH) twitching motility protein PilH [Type IV pili (VF0082)] [Pseudomonas aeruginosa PAO1]
k141_394847	fleQ	(fleQ) transcriptional regulator FleQ [Flagella (VF0273)] [Pseudomonas aeruginosa PAO1]
k141_410255	algR	(algR) alginate biosynthesis regulatory protein AlgR [Alginate (VF0091)] [Pseudomonas aeruginosa PAO1]
k141_425127	pilU	(pilU) twitching motility protein PilU [Type IV pili (VF0082)] [Pseudomonas aeruginosa PAO1]
k141_443234	fliP	(fliP) flagellar biosynthetic protein FliP [Flagella (VF0273)] [Pseudomonas aeruginosa PAO1]
k141_443234	fliQ	(fliQ) flagellar biosynthetic protein FliQ [Flagella (VF0273)] [Pseudomonas aeruginosa PAO1]
k141_445882	algC	(algC) phosphomannomutase AlgC [Alginate biosynthesis (CVF522)] [Pseudomonas aeruginosa PAO1]
k141_477016	fliP	(fliP) flagellar biosynthetic protein FliP [Flagella (VF0273)] [Pseudomonas aeruginosa PAO1]
k141_477379	fliM	(fliM) flagellar motor switch protein FliM [Flagella (VF0273)] [Pseudomonas aeruginosa PAO1]
k141_492959	flgH	(flgH) flagellar L-ring protein precursor FlgH [Flagella (VF0273)] [Pseudomonas aeruginosa PAO1]
k141_494604	flgC	(flgC) flagellar basal-body rod protein FlgC [Flagella (VF0273)] [Pseudomonas aeruginosa PAO1]
k141_50664	algC	(algC) phosphomannomutase AlgC [Alginate biosynthesis (CVF522)] [Pseudomonas aeruginosa PAO1]
k141_524666	fliQ	(fliQ) flagellar biosynthetic protein FliQ [Flagella (VF0273)] [Pseudomonas aeruginosa PAO1]
k141_524666	fliP	(fliP) flagellar biosynthetic protein FliP [Flagella (VF0273)] [Pseudomonas aeruginosa PAO1]
k141_528996	waaF	(waaF) heptosyltransferase I [LPS (VF0085)] [Pseudomonas aeruginosa PAO1]
k141_539669	fliM	(fliM) flagellar motor switch protein FliM [Flagella (VF0273)] [Pseudomonas aeruginosa PAO1]
k141_593211	algB	(algB) two-component response regulator AlgB [Alginate (VF0091)] [Pseudomonas aeruginosa PAO1]
k141_593475	pilI	(pilI) twitching motility protein PilI [Type IV pili (VF0082)] [Pseudomonas aeruginosa PAO1]
k141_599056	pvdH	(pvdH) diaminobutyrate-2-oxoglutarate aminotransferase PvdH [pyoverdine (IA001)] [Pseudomonas aeruginosa PAO1]
k141_64492	dotU1	(dotU1) type VI secretion system protein DotU [HSI-I (VF0334)] [Pseudomonas aeruginosa PAO1]
k141_64492	hsiJ1	(hsiJ1) type VI secretion system hcp secretion island protein HsiJ1 [HSI-I (VF0334)] [Pseudomonas aeruginosa PAO1]
k141_73625	pilH	(pilH) twitching motility protein PilH [Type IV pili (VF0082)] [Pseudomonas aeruginosa PAO1]
k141_79819	pilT	(pilT) twitching motility protein PilT [Type IV pili (VF0082)] [Pseudomonas aeruginosa PAO1]
k141_84914	flgG	(flgG) flagellar basal-body rod protein FlgG [Flagella (VF0273)] [Pseudomonas aeruginosa PAO1]

a VFDB, virulence factor database.

### Metagenome-assembled genomes.

**FIG 9:**
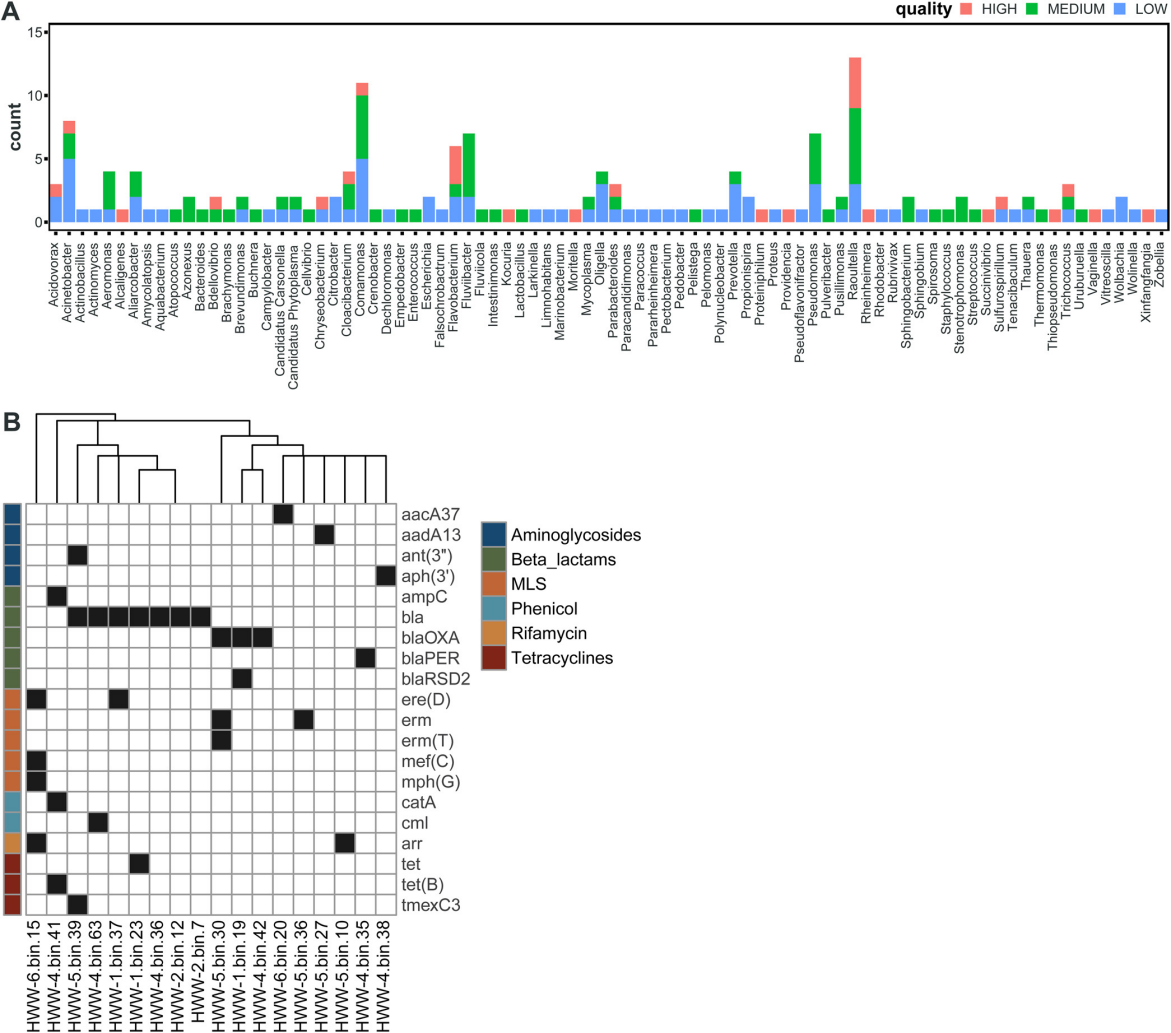
Metagenome-assembled genomes. (A) Histogram of MAG species assignment, colored by MAG quality. (B) ARG content of high-quality MAGs identified by AMRFinder.

To better study the content of individual genomes and the genetic contexts of ARGs, we constructed metagenome-assembled genomes (MAGs). We recovered a total of 167 draft MAGs of various quality: 26 high-quality, 65 medium-quality, and 76 low-quality drafts (Fig. 9A). The most commonly recovered genera were *Raoutella* (13/167), *Comamonas* (11/167), and Acinetobacter (8/167), all *Proteobacteria*. Of the total 84 unique genera identified, 54 were unique to just 1 MAG. Next, we screened the 26 high-quality MAGs for known ARGs *in silico*. We identified a total of 20 unique ARGs in 18/26 MAGs (Fig. 9B). Most of these MAGs (12/18) encoded at least one beta-lactamase, including *bla_OXA_* carbapenemase and *bla_PER_* extended-spectrum β-lactamases (ESBLs).

## DISCUSSION

We have shown that Indian HWW samples have a high abundance and diversity of clinically relevant ARGs potentially hosted by high-priority pathogens. We collected samples from six tertiary-care hospitals in north India with various patient loads, located in urban (HWW-1, -2, -4, and -5) and rural areas (HWW-3 and -6) (Fig. 1 and Table 1). Earlier studies have been limited to single hospitals (48). We used shotgun metagenomics to analyze our HWW samples, which has the advantage of quantifying thousands of genes from culturable as well as nonculturable taxa simultaneously. PCR-based approaches may have greater sensitivity to low-abundance ARGs due to targeted amplification (49), but they are limited in the ARGs able to be detected. For example, a study comparing culture-based approaches and metagenomic methods showed that the culture-based technique isolated bacteria from 104 out of 539 clinical samples and therefore, captured resistance against 8 antibiotic types in only 16.17% of the clinical samples. Contrary to the culture method, metagenomic analysis identified 1,573 species and 885 ARG subtypes in the hospital sewage (50).

Hospital wastewater surveillance is useful for monitoring antibiotic-resistant bacteria and the ARG load in the hospital environment (51). In our study, we found high ARG richness (fragments per kilobase million [FPKM]) in hospital wastewater. A study reported a comparably lower abundance of ARGs in urban wastewater compared to our findings (11). In India, hospital wastewater treatment is inadequate and the hospital wastewater mostly gets released into the public sewer network increasing the probability of ARG dissemination to humans, especially to the poor who often reside near the drains (22, 51).

Owing to its high abundance, the phyla *Proteobacteria* carried most of the ARGs, constituting approximately 85% of the ARG abundance (range, 78% to 84%) in terms of FPKM, followed by *Bacteroidetes* (HWW-1 to HWW-4), *Firmicutes* (HWW-5), and *Actinobacteria* (HWW-6) (Fig. 3 to 5). The taxonomic compositions of our HWW samples are similar to that reported from a single hospital in the city of Mumbai, located in western India (48). They also reported the dominance of *Proteobacteria*, followed by *Bacteroidetes* and *Firmicutes*. Interestingly, they reported Acinetobacter as the dominant genus (30%), which is also true of our HWW samples from New Delhi (HWW-4 = 26% and HWW-5 = 18%); however, Acinetobacter was not among even the top three most abundant genera in the other samples (Fig. 2 and 3). Pseudomonas was among the top four genera (3% to 9%), carrying maximum ARG abundance (23% to 38%) in all the samples except HWW-4. In congruence with the aforementioned study, Acinetobacter predominantly carried 23% ARG abundance in HWW-4 of New Delhi (48). The species carrying most of the ARGs were P. aeruginosa across all the samples, *A. baumanii* in HWW-4, and P. putida in HWW-6. All these species are high priority, nosocomial pathogens with a high rate of ARG dissemination, mostly involved in hospital-acquired infections (52, 53). The opportunistic pathogens *A. baumanii* and P. aeruginosa carry natural intrinsic resistance toward multiple drug classes, including beta-lactamases and other carbapenemases. They are not eliminated by wastewater treatment either (54, 55). P. putida is an environmental Gram-negative bacterium. It is rarely a causative agent for human diseases, but there have been reports of serious infections and outbreaks from time to time. Sometimes it functions as an exchange platform for ARGs, spreading ARGs to more pathogenic species like P. aeruginosa (56).

We generally expected HWW from the largest hospitals to have the highest ARG burdens due to increased patient load. Surprisingly, the samples from hospitals with the largest number of beds (HWW-1 = 2,400 beds and HWW-2 = 1,050 beds) had among the lowest ARG abundances. Conversely, HWW-4 and HHW-6 had 4 to 6 times higher ARG abundance but with a fraction of the number of beds (HWW-4 = 650 and HWW-6 = 300). This could be due to improved antibiotic stewardship and waste disposal practices. However, we note that “hospital size” is not definitive because many hospitals in India over admit 3 to 4 times the total bed capacity, and these true numbers may not be reported (22).

Several studies have reported the injudicious use of broad-spectrum antibiotics in Indian hospitals and, consequently, high incidences of beta-lactamase (carbapenemase) resistance (57, 58). We also detected predominant resistance against broad-spectrum antibiotics with a leading abundance of aminoglycoside resistance in all the samples except HWW-1 and HWW-3 where macrolide resistance was most abundant (Fig. 4A and 5) (48). Carbapenem and sulfonamide resistance was among the top five ARG-enriching drug classes in all the samples. Earlier, in Indian HWW, the *sul1* sulfonamide resistance gene was reported as the most abundant (11.4%); we also identified *sul1* in all HWW samples but it was among the top 10 most abundant ARGs only in HWW-2 and HWW-6 (2.2% and 9.6%, respectively) (Fig. 4) (48). The ARG composition listed in the top 10 ARGs replicated the abundance of resistance evaluated at the drug class level with 8 aminoglycoside resistance genes, 6 macrolide resistance genes, and 3 carbapenemases. Among the top 10 ARGs, the beta-lactamases *bla*_OXA-10_, *bla*_GES-1_, and *bla_RSA-1_* were dominant. Multiple incidences of *bla*_OXA_ outbreaks in Indian hospitals have been reported in the past, which correlates with *bla*_OXA_ being the most abundant carbapenemase in all our samples (59, 60). The *bla*_OXA-10_ and GES-type ESBLs have been earlier reported as the most abundant beta-lactamase in one of the Mumbai hospitals (48). The *bla*_NDM-1_ is endemic to India and is often flanked by transposable elements. The *bla*_NDM-1_ was found prevalent among P. aeruginosa, K. pneumoniae, and *A. baumanii* (Fig. 6A to C). The use of colistin used as the last-resort antibiotic in extreme clinical cases of MDR and extensively drug-resistant (XDR) infections also leads to the emergence of *mcr* variants. The detection of plasmid-mediated *mcr-5.1* in HWW-6 (Fig. 6D) for the first time in Indian hospital sewage raises concern for future healthcare systems and is an alarming signal for the upcoming antibiotic apocalypse when no antibiotic will work. MAGs construction and analysis also revealed at least one beta-lactamase, including *bla_OXA_* carbapenemase and *bla_PER_* ESBLs. Most of the ARGs identified in our study can efficiently transmit to different species of bacteria through horizontal gene transfer, and hence, their spread to the local population has become a challenge for healthcare workers. The concentration of several ARGs like blaOXA-1, blaOXA-10, and blaTEM-1 increases with wastewater treatment procedures (61). Plasmid carried the maximum ARGs in all the samples reflecting the high probability of AMR spread through hospital wastewater.

In this study, we identified several virulence factors mostly associated with the general secretion pathway, motility, and alginate biosynthesis often involved in biofilm formation (Table 3). These VFs aid the innate resistance against antibiotics (45, 47, 62).

Our study adds further evidence that India is facing an “AMR pandemic” and needs urgent national surveillance for assessing the ARG risk. Hospital wastewater transports high-risk clinical threats to the public sewer system and facilitates their dissemination to the public through HGT (61). The common practice of open drainage systems and inadequate sanitation measures in most parts of India (63) may lead to infection spread with antibiotic-resistant bacteria and outbreaks in the community as well as a hospital setting. Likely due to our limited sample size, we did not find significant dissimilarities in ARGs and microbial diversity with geographical variations. Overall, the ARG diversity in our samples was in concordance with one of the earlier studies on Indian hospital wastewater samples, carried out in Mumbai (48).

This explorative study shows that Indian HWW contains an abundance of high-risk ARGs that are present in mobile genetic elements and are carried by high-priority pathogens. Antimicrobial risk management strategies should be immediately implemented in hospitals. Efficient wastewater treatment strategies meeting international standards should be implemented in hospitals, as well as areas downstream including the public sewage systems where the HWW are deposited.

## MATERIALS AND METHODS

### Sample collection and processing.

Wastewater samples were collected from six hospitals located in different regions of northern India from December 21, 2019, to March 21, 2021 (Table 1). From the main sewage pipeline receiving effluents from every other pipeline in the hospital, multiple samples were collected from adjacent points in sterile bottles and pooled into one sample of 2 liters of unfiltered sewage water. Samples were stored on ice without additives or DNA stabilizers. In a sterile environment, samples were vortexed and 50 mL was collected and then centrifuged again at 7,000 × *g* to separate cell pellets and water. The pellet was stored at –20°C until DNA extraction.

DNA was extracted using the DNeasy PowerSoil kit (Qiagen) as per the manufacturer’s instructions. Extracted DNA was quality checked using NanoDrop 2000 Spectrophotometer (ThermoFisher Scientific), to check for RNA and protein contaminants. Further, to validate the quantitative estimation of the extracted DNA, 50 ng of extracted DNA and Lambda DNA/HindIII Marker (SM0102; ThermoFisher Scientific) was loaded on a 1% agarose gel stained with ultrapure ethidium bromide (ThermoFisher Scientific) and electrophoresed by running the gel at 80 V for 1 h. Finally, the gel was imaged in a SmartView Pro 1100 Imager System (Major Science). Sample DNA concentrations were quantified using Qubit dsDNA HS assay kit (Thermo Fisher Scientific), as per manufacturer’s instructions, using a Qubit 3.0 Fluorometer (Thermo Fisher Scientific) (64).

### DNA library preparation and sequencing.

One hundred nanograms of intact DNA was enzymatically fragmented using Covaris targeting the 250-bp fragment size, followed by end repair to convert the overhangs into blunt ends. To the adenylated fragments, loop adapters were ligated and cleaved with a uracil-specific excision reagent (USER) enzyme. The samples were further purified using AMPure beads. DNA was enriched by PCR with six cycles using NEBNext Ultra II Q5 master mix, Illumina universal primer, and sample-specific octamer primers. The amplified products were cleaned by using AM pure beads to remove unused primers. The final DNA libraries were eluted in 15 mL of 0.1× TE buffer, and concentrations were quantified using the Qubit DNA HS assay kit and a Qubit.3 Fluorometer.

Library quality was assessed using DNA 5000 ScreenTape in an Agilent 4150 Tape Station system. Here, 1 mL of the library was mixed with 5 mL sample buffer, vortexed, and then spun to collect the sample to the bottom of the strip. The strip was then loaded into the Agilent 4150 Tape Station instrument. The library qualification criteria were the presence of a broad peak in the range of 200 bp to 1,000 bp, with an average size of 350 bp in the Agilent 4150 TapeStation system, the Qubit concentrations above 2 ng/μL or 10 nmol/L and library devoid of primer, adapter, and larger size peaks.

The quality-passed libraries were diluted to 2 nM and pooled. We then performed shotgun metagenome sequencing on the pooled libraries using Illumina HiSeq, 2 × 150 bp paired-end run. The sequence reads were demultiplexed by barcode using bcl2fastq v2.1.9. Sequence data quality was checked using FastQC v0.11.9 (65) and MultiQC v1.9 (66) for base call quality distribution, percent bases above Q20, Q30, percent GC, and sequencing adapter contamination. The number of reads sequenced (in million) was approximately 54, 45, 45, 78, 43, and 43 for HWW-1, HWW-2, HWW-3, HWW-4, HWW-5, and HWW-6, respectively.

For microbiome composition, and contig-based ARG analyses, these clean reads were assembled using MEGAHIT v1.2.9 (67) with *–k-min 35 –k-max 141 –k-step 28* parameters. The contigs shorter than 200 bp were removed from further analysis. Assembly quality was checked using Bowtie2 v2.1.0 (68).

### Taxonomic classification.

For taxonomic classification, the reads were quality filtered using fastp v.0.20.1 (69). These clean reads were taxonomically classified using Kraken2 with the NCBI nonredundant nucleotide database as a reference (70). Kraken hits with a relative abundance of <0.1% reads were filtered out.

### Identification and quantification of ARG.

For ShortBRED analyses, reads were quality filtered using Trimmomatic v0.38 (71) with the following parameters: *ILLUMINACLIP: NexteraPE-PE.fa:2:30:10:1:true SLIDINGWINDOW:4:20 LEADING:10 TRAILING:10 MINLEN:60*. Clean read quality was assessed using FastQC v0.11.7 (65) and MultiQC v1.2. (66). ARG abundances were quantified using ShortBRED v0.9.4 (25). We built an ARG-specific markers database from 7,921 antibiotic resistance proteins using *‘shortbred_identify.py’* with the following nondefault parameters: –*clustid* 0.95 –*ref* Uniref90 (72). The antibiotic resistance protein sequences include sequences from the CARD database (30), the NCBI-AMR database (73), and antibiotic resistance proteins identified using functional metagenomics in this cohort, and previous studies (74–82). These AMR gene families were then manually curated, and entries with the following criteria were removed because they would not be confidently expected to provide resistance based solely on a short-read marker:

1. Genes associated with global gene regulators, two-component system proteins, and signaling mediators (e.g., blaZ, vanS-vanR, mecI, mepR, gadW, marR);
2. Genes encoding subunits that are part of multiple efflux pumps (e.g., tolC, oprM, opmD);
3. Resistance via mutation in genes (e.g., resistance to antifolate drugs via mutations in dhfr, resistance to rifamycin via mutation in rpoB);
4. Genes conferring resistance by modifying cell wall charge (e.g., mprF);
5. Genes that reduce permeability (e.g., omp38, tmrB) or confer resistance through overexpression (e.g., thymidylate synthase); and
6. General efflux pumps that came through functional selections (e.g., MFS-type, ABC-type).

The relative abundance of AMR gene families was quantified by mapping reads to the filtered set of marker sequences using *shortbred_quantify.py*. ShortBRED hits were filtered out if they had counts less than 2 or a mean RPKM < 0.001.

For contig-based ARG analyses, the reads were assembled using MEGAHIT v1.2.9 (67) with the following parameters: *–k-min 35 –k-max 141 –k-step 28*. Contigs less than 200 bp were removed from the downstream analysis. The assembly quality was checked using Bowtie2 v2.1.0 (68). Contigs were assigned taxa using TaxonKit v0.2.3 (83) and the NCBI nucleotide database, and those annotated as Eukaryotic sequences were filtered out. The remainder were annotated using Prokka v1.14.6 *–metagenome* (84). ARGs were called using the Resistance Gene Identifier (RGI) v5.2.0 and the Comprehensive Antibiotic Resistance Database (CARD) v3.1.1 (https://github.com/arpcard/rgi) (30). Sequences were called as an ARG if they had ≥80% coverage and ≥90% identity to a reference ARG. Sequences with 100% coverage and 100% identity against a reference ARG were classified as “Perfect” hits; those with <95% identity but >65% coverage as putative or potential ARGs. Additionally, fARGene (Fragmented Antibiotic Resistance Gene iENntifiEr) (https://github.com/fannyhb/fargene) was used to identify gene fragments in contigs. ARG abundances were quantified as FPKM (11):

$$\mathrm{FPKMi}\text{ = }\mathrm{qi}/\left(\mathrm{li}\times Q\right)\times 10^{6}$$

where *q_i_* = no. of reads mapped to the contig, *l_i_* = length of contig, and *Q* = total no. of mapped reads.

Taxonomic distribution of BLAST hit contigs was identified with TaxonKit v0.2.3. When the ARG containing contig was simultaneously annotated against the CARD database and a microbial taxon in the NCBI NR database, we considered that the associated microbial taxon was the carrier of the corresponding ARG (44). ARGs were identified as located in plasmids or chromosomes using PlasClass and PlasFlow v1.1 (85, 86). Virulence factors were identified using ABRicate v1.0.1 (https://github.com/tseemann/abricate) (Table 3).

### Metagenome-assembled genomes.

To extract MAGs, metagenomic assemblies were generated using MEGAHIT v1.1.4 with *–min-contig-len 1000*. The reads were mapped back to their assembly using Bowtie2 v2.3.5, then converted to the BAM format with SAMtools v1.9 (87). Single-sample metagenomic binning was applied using MetaBAT v2.11.2 with options *–minContigLength 1500*, producing 215 bins total. MAG quality was assessed using BBMap v38.82, QUAST v4.5 (88), and CheckM v1.0.7 (89). Quality was scored on the basis of the following criteria: high-quality draft: completion >90%, contamination <5%; medium-quality draft: completion ≥50%, contamination <10%; and low-quality draft: completion <50%, contamination <10%. Forty-eight samples did not meet these requirements (i.e., contamination ≥10%) and were excluded. The MAG taxonomy was assigned using Mash v2.2 (90) with a mash sketch of the NCBI RefSeq database (accessed 25 May 2021), reporting the genus of the top-scoring hit. MAGs were screened for known ARGs using AMRFinder v3.9.8 (73).

### Statistical analysis.

All the statistical analysis has been done in R 4.1.2 using packages like vegan, cowplot, ggplot, pheatmap, phyloseq, ggpubr, and ggvegan (91). The Spearman’s correlation for ARG-taxa cooccurrence network was calculated in Jamovi v2.2. and was plotted in Cytoscape v3.9.1 (92, 93).

### Ethics approval and consent to participate.

Not applicable. Preapproval from the ethical committee is not required in India to work on hospital sewage samples.

### Data availability.

All metagenomic sequencing data are available at the National Center for Biotechnology Information (NCBI) database with BioProject accession number PRJNA682952 and SRA accession numbers SRR13227005, SRR13227004, SRR13227003, SRR13227002, SRR15384560, and SRR15384559.
